# A systematic characterization of amino acid metabolism–related genes reveals molecular subtypes and a prognostic signature in bladder cancer

**DOI:** 10.3389/fonc.2026.1754912

**Published:** 2026-02-27

**Authors:** Junrui He, Xu Liu, Shirui Li, Zhongyou Xia, Xiaojun Tan, Lijuan Peng, Qiongxian Long, Ji Wu

**Affiliations:** 1 Department of Urology, The Affiliated Nanchong Central Hospital of North Sichuan Medical College, Nanchong, Sichuan, China; 2 Department of Urology, The Second Clinical Medical College of North Sichuan Medical College, Nanchong, Sichuan, China; 3 Department of Urology, Beijing Anzhen Nanchong Hospital, Capital Medical University & Nanchong Central Hospital, Nanchong, Sichuan, China; 4 Department of Pathology, Beijing Anzhen Nanchong Hospital, Capital Medical University & Nanchong Central Hospital, Nanchong, Sichuan, China

**Keywords:** amino acid metabolism, bladder cancer, machine learning, molecular subtype, prognostic signature, PSPH

## Abstract

**Background:**

Amino acid metabolism is integral to tumor proliferation, redox control, and immune regulation. Yet, studies in bladder cancer have largely centered on single amino acids, leaving the broader metabolic gene network insufficiently characterized.

**Methods:**

Transcriptomic and clinical data from TCGA-BLCA, GSE13507, and GSE32894 were integrated with 32 MSigDB amino acid metabolism gene sets. Differential analysis, enrichment profiling, and consensus clustering defined metabolic subtypes. WGCNA and survival filtering identified candidates for a prognostic model, which was optimized using the MIME platform. Immune features and drug sensitivities were evaluated through multiple deconvolutions and pharmacogenomic resources. Single-cell data (GSE222315) were used to trace the cellular origin of model genes. *PSPH* expression and function were validated in tissues and bladder cancer cell lines.

**Results:**

A total of 144 dysregulated amino acid metabolism–related genes were identified and used to define two distinct metabolic subtypes. One subtype was marked by coordinated upregulation of glutamine, branched-chain amino acid, tryptophan, and serine metabolic programs, accompanied by higher grade and stage, significantly worse survival, and dense but functionally impaired immune infiltration. From 24 candidate genes, a 16-gene metabolic signature was constructed and consistently validated across TCGA, GSE13507, and GSE32894, showing strong and stable prognostic performance superior to several published models. High-risk group displayed activation of cell-cycle, DNA-replication, *mTORC1*, and inflammatory-stress pathways, together with predicted sensitivity to *PI3K/mTOR* inhibitors, DNA-damaging agents, and selected epigenetic or cytoskeletal drugs. In the IMvigor210 cohort, the high-risk group showed a greater likelihood of responding to *PD-1/PD-L1* blockade. Single-cell profiling localized signature expression predominantly to malignant epithelial cells. *PSPH*, a core model gene, was overexpressed in tumor tissues and cell lines, and functional assays demonstrated its role in promoting proliferation, migration, invasion, and survival of bladder cancer cells.

**Conclusions:**

This study highlights the central role of amino acid metabolic networks in shaping bladder cancer heterogeneity and provides a metabolically grounded framework for risk stratification and therapeutic development.

## 1 Introduction

Bladder cancer (BCa) ranks as the ninth most prevalent cancer worldwide, with approximately 614,000 new cases and 220,000 deaths reported in 2022. Men exhibit significantly higher age-standardized incidence and mortality rates compared to women, with rates of 9.3 and 3.1 per 100,000, respectively, whereas women have rates of 2.4 and 0.8 per 100,000 (1). Despite the availability of conventional treatments such as radical surgery, intravesical therapies, platinum-based chemotherapies, and immune checkpoint inhibitors, challenges remain, including high recurrence rates, rapid disease progression, and significant biological heterogeneity, which hinder long-term survival outcomes (2–6). Additionally, immune checkpoint inhibitors provide benefits to only a small subset of patients, and reliable biomarkers for predicting treatment efficacy and prognostic outcomes remain difficult to identify (7–9). These issues underscore the urgent need for advanced molecular classifications and effective prognostic tools.

Metabolic reprogramming is a hallmark of cancer, facilitating the uncontrolled growth of malignant cells while also influencing the tumor microenvironment and modulating immune responses (10–12). A key aspect of this metabolic reprogramming is amino acid metabolism, which plays an essential role in energy production, maintaining redox balance, and regulating immune cell activity (13, 14). Previous studies have shown that alterations in amino acid metabolic pathways, such as glutamine dependence, tryptophan metabolism, and branched-chain amino acid degradation, are closely linked to tumor development, immune evasion, and therapeutic resistance across various cancers (15–20). However, the distribution and clinical significance of amino acid metabolism-related genes (AAMRGs) in BCa have not been thoroughly investigated.

Research has demonstrated that BCT-100 induces autophagy and apoptosis associated with arginine depletion via ROS-mediated inhibition of the *AKT/mTOR* signaling pathway (21). Furthermore, the competitive interaction between *p53* and *YY1* in regulating *PHGDH* is crucial for modulating serine metabolism and promoting BCa progression, highlighting the significance of reprogramming amino acid metabolism in this disease (22). Risk assessment models focusing on the glutamine and tryptophan pathways have received preliminary support from independent cohorts and *in vitro* studies (23–27). However, current investigations predominantly concentrate on specific localized pathways or a limited set of genes, which restricts our understanding of how the synergistic imbalance among various amino acid metabolic pathways impacts tumor metabolism and immune diversity.

To address these gaps, we performed an integrative multi-cohort analysis of AAMRGs in BCa. Using a precision-subtyping perspective, we identified metabolism-informed subtypes and assessed their relationships with clinical outcome, the immune microenvironment, and immunotherapy-related profiles (28). A subtype-derived prognostic signature was subsequently constructed and validated in independent datasets, with additional support from single-cell transcriptomics, clinical specimens, and functional assays (29). This metabolism-centered framework enables molecular classification and risk stratification in BCa while highlighting candidate targets for future exploration.

## 2 Materials and methods

### 2.1 Data acquisition

Transcriptomic, somatic mutation, and clinical data for BLCA were obtained from The Cancer Genome Atlas (TCGA) using the easyTCGA R package. GSE13507, GSE32894, and single-cell dataset GSE222315 were downloaded from the Gene Expression Omnibus (GEO) database (https://www.ncbi.nlm.nih.gov/geo/). Thirty-two amino acid metabolism-related gene sets were curated from MSigDB (https://www.gsea-msigdb.org/gsea/msigdb) by combining the search terms amino acid and metabolism.

Expression and mutation data for human cancer cell lines were retrieved from the Cancer Cell Line Encyclopedia (CCLE; https://portals.broadinstitute.org/ccle/). CERES scores from genome-scale CRISPR screens (18,333 genes across 739 lines) were obtained from the Dependency Map portal (DepMap; https://depmap.org/portal/). Drug sensitivity data were sourced from the Cancer Therapeutics Response Portal (CTRP v2.0; https://portals.broadinstitute.org/ctrp/) and the PRISM Repurposing dataset (19Q4 release; https://depmap.org/portal/data_page/). Transcriptomic and clinical data for the IMvigor210 cohort were accessed through the IMvigor210CoreBiologies package.

For TCGA-BLCA, one “1A”-labeled sample per patient (barcode positions 15–16) was retained to exclude intra-patient duplicates. Differential expression was performed on raw counts using DESeq2, while TPM-normalized data were used for all downstream analyses, including limma-based tests. Samples with survival under 30 days were excluded from DSS analysis to reduce perioperative or non-cancer-related bias.

### 2.2 Enrichment analysis

Multiple enrichment strategies were employed to investigate the functional roles of differentially expressed genes (DEGs). GO and KEGG over-representation analyses were performed using the clusterProfiler package, with significance evaluated by hypergeometric testing and corrected via the Benjamini–Hochberg method. Pathways with a false discovery rate (FDR) below 0.05 were considered significant. GSEA was conducted using limma-derived genes (adjusted p< 0.05), ranked by log2 fold change against KEGG gene sets from MSigDB. Sample-level pathway activity was quantified by ssgsea using the GSVA package, and group differences were assessed via limma (adjusted p< 0.05).

### 2.3 Immune infiltration analysis

Immune cell infiltration was profiled using the IOBR package through four deconvolution algorithms (CIBERSORT, EPIC, MCPcounter, and quanTIseq), while the ESTIMATE algorithm provided immune and stromal scores as well as tumor purity (30). To evaluate immune functional activity, ssGSEA was applied to IOBR-integrated immune gene sets, generating sample-level enrichment scores. Group differences in infiltration or immune pathway activity were tested using iobr_cor_plot function, which automatically applies Wilcoxon or Kruskal–Wallis tests based on group number. Multiple testing correction was performed via the Benjamini–Hochberg method, with significance set at FDR< 0.05.

### 2.4 Molecular subtyping analysis

Molecular subtypes were identified via consensus clustering on genes overlapping between differentially expressed and amino acid metabolism–related genes. The ConsensusClusterPlus package was used with k-means, Euclidean distance, K set to 6, and 1000 iterations (resampling 80% of samples each time) (31). The optimal cluster number was selected based on CDF curves, consensus heatmaps, silhouette width, PAC index, and biological interpretability. Principal component analysis (PCA) was used for visualization, and survival differences among subtypes were evaluated using Kaplan–Meier curves with log-rank tests. These subtypes reflected intratumoral heterogeneity and supported downstream analyses of clinical, immune, and metabolic features.

### 2.5 Weighted gene co-expression network analysis

Weighted gene co-expression network analysis (WGCNA) was performed on transcriptomic data from tumor and adjacent normal tissues to identify bladder cancer–specific gene modules (32). Outlier samples were excluded based on hierarchical clustering. The optimal soft threshold was determined by assessing scale-free topology (R² = 0.9) and mean connectivity. An unsigned weighted network was built, modules were defined via dynamic tree cutting, and merged by eigengene similarity. Module–trait relationships were evaluated using “tumor vs. normal” as the phenotype, and modules significantly correlated with tumor tissue (P< 0.05) were selected for downstream analysis.

### 2.6 Construction of an amino acid metabolism–related risk score model

A prognostic risk model was developed using the MIME R package based on amino acid metabolism–related genes and survival data (33). MIME integrates 10 classical survival algorithms and generates 117 model combinations, automatically evaluated by cross-validation. The model with the highest mean C-index and time-dependent AUC was selected as optimal. Risk scores were calculated as a weighted sum of normalized gene expression and corresponding regression coefficients using the following formula:


$$\text{RiskScore}=\left(\sum_{\text{i}=1}^{\text{n}}{\text{β}}_{\text{i}}\times \text{Expressio}{\text{n}}_{\text{i}}\right)$$

where $\beta_{i}$ is the regression coefficient for gene i, and ${\text{Expression}}_{i}$ is its normalized expression level. Patients were stratified into high- and low-risk groups based on the median score, following prior literature.

Univariate Cox regression was applied in both the training set (TCGA-BLCA) and external validation cohorts (GSE13507 and GSE32894), followed by a fixed-effects meta-analysis to combine hazard ratio (HR). Forest plots were generated to show effect consistency. Several published prognostic signatures were benchmarked across datasets, and their HR and time-dependent AUC (1, 3, and 5 years), along with Harrell’s C-index, were compared. The final model demonstrated stable, generalizable predictive performance and potential advantages over existing signatures.

### 2.7 Prognostic independence analysis and nomogram construction

The independent prognostic value of the risk score was evaluated via univariate and multivariate Cox regression using the survival package, alongside clinical variables such as age and tumor stage. Significant predictors were incorporated into a nomogram built with the rms package to estimate 1-, 3-, and 5-year survival probabilities. Model performance was assessed through calibration curves and decision curve analysis (DCA), reflecting predictive accuracy and clinical benefit, respectively. The concordance index (C-index) was used to compare the prognostic discrimination of the risk score alone versus the integrated nomogram, highlighting their relative utility in clinical outcome prediction.

### 2.8 Drug response prediction

To assess associations between the risk score model and drug response, pharmacogenomic data were retrieved from GDSC, CTRP, and PRISM. Drug sensitivity was quantified as IC50 (GDSC) or AUC (CTRP, PRISM), with lower values indicating greater sensitivity. IC50 values in GDSC were estimated using the oncoPredict package, and Spearman correlations were calculated between gene expression weighted by model coefficients and drug response, guided by known target pathways (34). In CTRP and PRISM, compounds with >20% missing AUCs were excluded, and remaining values imputed via the k-nearest neighbor (KNN) method (35). AUCs were predicted using calcPhenotype (pRRophetic) based on CCLE expression profiles, and intergroup comparisons were conducted to identify candidate therapeutics.

### 2.9 Immunotherapy response prediction

Immune activity across tumor samples was evaluated using the TIP framework (http://biocc.hrbmu.edu.cn/TIP/index.jsp), which quantifies seven steps of the cancer–immunity cycle based on ssgsea scores from curated gene sets. Group differences were assessed using non-parametric tests to characterize immune landscape and potential therapy response. The IMvigor210 cohort, containing transcriptomic and clinical data, was used for external validation. Patients were assigned risk scores based on model coefficients and stratified by the median. Associations between risk and response (CR, PR, SD, PD) were analyzed using Kruskal–Wallis and Fisher–Freeman–Halton tests. Lastly, SubMap analysis via GenePattern (https://cloud.genepattern.org/gp/pages/index.jsf) was applied to align expression profiles from bladder cancer risk groups with IMvigor210 responders, estimating PD-L1 sensitivity and offering transcriptome-based evidence to guide immunotherapy (36).

### 2.10 Single-cell transcriptomic analysis

Single-cell transcriptomic analysis was conducted using the GSE222315 dataset, comprising nine tumor and matched normal bladder samples. A Seurat v5 object was constructed and processed through standard quality control (37). Cells with low feature counts, high mitochondrial or ribosomal content, or erythroid signatures were removed; high-quality cells met thresholds for gene number (200–6000), transcript counts (<50,000), and percent.mt (<20%). Cell cycle states were annotated and regressed out during scaling. PCA was performed on 2,000 variable genes, with batch correction via Harmony. Dimensionality reduction used uniform manifold approximation and projection (UMAP) and t-distributed stochastic neighbor embedding (t-SNE) (top 20 PCs), and clustering was achieved with Louvain (resolution = 0.5). Twenty clusters were annotated into eight major cell types. Gene-level and module-level expression of model features were visualized across clusters and tissues. Metabolic activity differences, computed via AddModuleScore, were compared between tumor and normal tissues using the Kruskal–Wallis test.

### 2.11 Clinical samples immunohistochemistry

Tumor and adjacent bladder tissues were collected from patients with primary urothelial carcinoma who underwent radical cystectomy. Patients were required to have no history of systemic chemotherapy, radiotherapy, or transurethral tumor resection for at least six months before surgery to avoid treatment-related alterations in tissue morphology and protein expression. Adjacent tissues were sampled at a distance greater than 2 cm from the visible tumor edge and were confirmed histologically to be free of malignant involvement. Immunohistochemical staining was performed on FFPE sections, and protein expression was evaluated using a semi-quantitative immunoreactivity scoring system incorporating staining intensity and the proportion of positive cells. According to the total score, cases were classified as low (≤4) or high (>4) expression. Detailed staining procedures and scoring criteria are provided in the **Supplementary Methods**.

### 2.12 *In vitro* experiments

Transcriptional and protein expression profiles of the candidate genes were examined in the normal urothelial cell line SV-HUC-1 and the BCa cell lines 5637 and T24 using quantitative PCR and Western blotting. The gene selected for subsequent functional investigation was determined by integrating evidence from previous studies, differential-expression findings obtained in earlier analytical stages, and expression variations observed in the aforementioned cell-based assays. For gain- and loss-of-function analyses, plasmids enabling overexpression or knockdown were generated and transiently introduced into 5637 and T24 cells, and transfection efficiency was verified by quantitative PCR and Western blotting. Functional assessments included immunofluorescence staining, CCK-8 proliferation assays, wound-healing migration assays, Transwell invasion assays, and flow-cytometric analysis of cell-cycle distribution and apoptosis. Detailed experimental procedures and reagent information are provided in the **Supplementary Methods**.

### 2.13 Statistical analysis

All analyses were primarily performed in R (v4.4.4), with additional evaluations and image quantification conducted in GraphPad Prism (v10.6) and ImageJ (v1.5.4). Continuous variables were examined for normality and homogeneity of variance before analysis. Normally distributed data were compared using unpaired or paired t-tests, one-way ANOVA, or two-way ANOVA as appropriate. For non-normal data, the Wilcoxon rank-sum test or Wilcoxon signed-rank test was applied for two-group comparisons, and the Kruskal–Wallis test was used for comparisons involving more than two groups. Categorical variables were analyzed using the chi-square test, Fisher’s exact test for 2×2 tables, the Fisher–Freeman–Halton test for multi-category comparisons, and McNemar’s test for paired categorical data. Correlation analyses used Pearson or Spearman coefficients depending on data distribution. Survival differences were assessed with Kaplan–Meier curves and log-rank tests, and prognostic factors were evaluated using univariate and multivariate Cox regression models. All tests were two-sided, with P< 0.05 considered statistically significant. Figure annotations followed the convention: ns (not significant), * or # for P< 0.05, ** or ## for P< 0.01, *** or ### for P< 0.001, and **** or #### for P< 0.0001.

## 3 Results

### 3.1 Molecular subtype identification of bladder cancer

**Figure 1:**
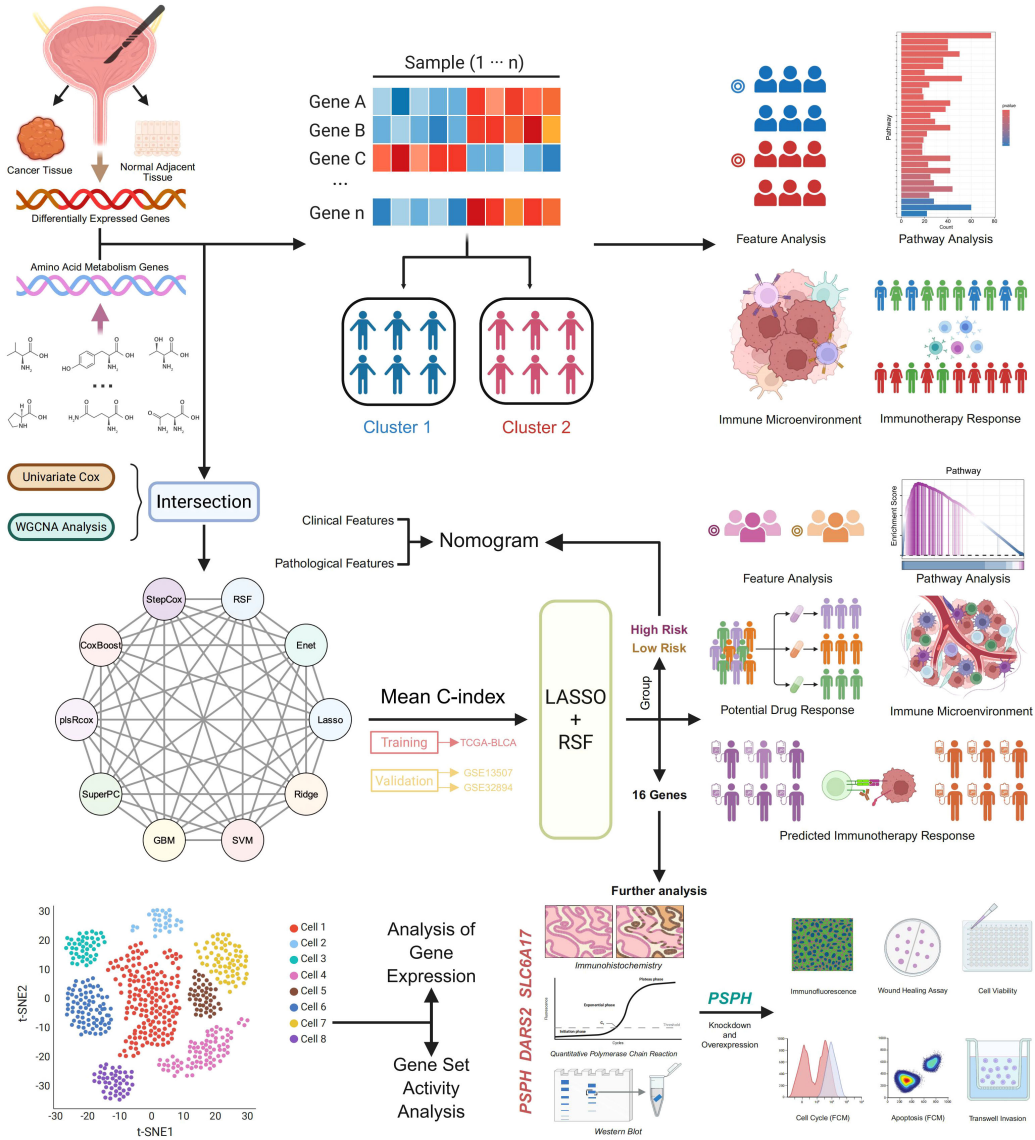
Workflow of the research study.

**Figure 2:**
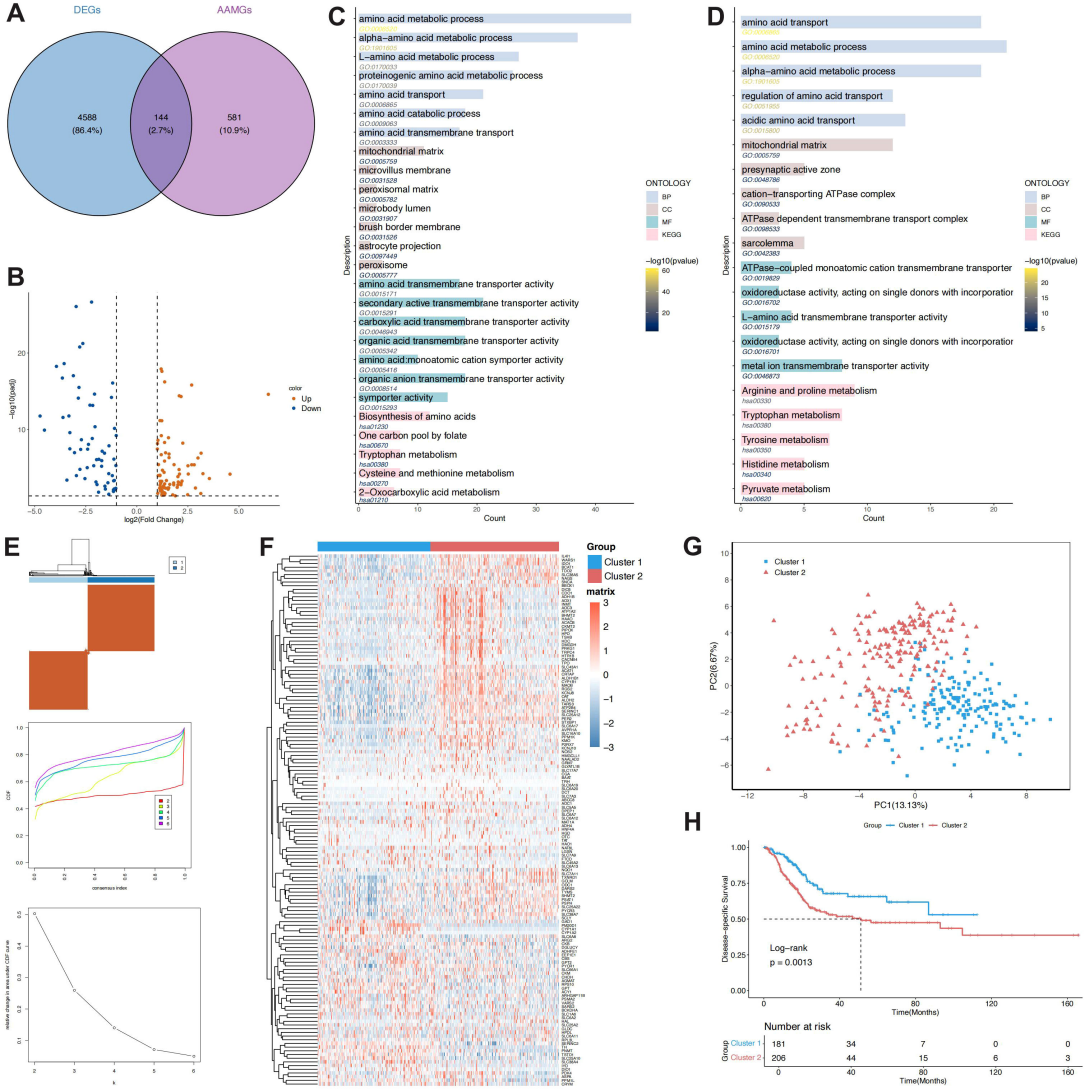
Amino acid metabolism-related gene enrichment analysis and molecular clustering Identification. **(A, B)** Genes derived from the intersection of differential genes and amino acid metabolism-related genes. **(C)** GO and KEGG enrichment analysis of upregulated genes. **(D)** GO and KEGG enrichment analysis of downregulated genes. **(E)** Confirmation of clustering results based on heatmap, CDF, and delta values. **(F)** Gene expression differences across molecular subtypes. **(G)** PCA analysis of clustering results. **(H)** Survival analysis across subtypes.

**Figure 1** illustrates the workflow of this study. In TCGA data, 4,732 DEGs (filtered with |logFC| > 1 and FDR< 0.05) overlapped with 725 AAMGs, yielding 144 genes—83 upregulated and 61 downregulated compared to pooled adjacent normal tissues (**Figures 2A, B**). GO and KEGG analyses showed that upregulated genes were enriched in amino acid catabolism and energy pathways, including arginine, proline, glutamate, and alanine metabolism, with primary localization in the cytoplasm and mitochondria (**Figure 2C**). This suggests that the elevated expression of these genes supports tumor growth by facilitating substrate acquisition and maintaining redox balance, key aspects of metabolic reprogramming. Conversely, downregulated genes, associated with basic metabolic functions such as alpha-amino acid metabolism and L-amino acid transport, were localized in mitochondria and peroxisomes. These genes play roles in core processes like amino acid biosynthesis and one-carbon metabolism, suggesting that essential metabolic activities may be suppressed in tumors (**Figure 2D**).

We performed unsupervised clustering with the 144 differentially expressed AAMGs to identify BCa molecular subtypes. Based on the consensus matrix heatmap, CDF curve, and the relative change in area under the curve for different K-values, two clusters were determined as optimal, reflecting both biological relevance and clustering performance (**Figure 2E**). The two subtypes, Cluster 1 and Cluster 2, were clearly distinguishable in the expression heatmap (**Figure 2F**) and further validated by PCA analysis (**Figure 2G**). Survival analysis revealed significantly poorer outcomes for patients in Cluster 2 compared to Cluster 1 (p = 0.0013, **Figure 2H**), suggesting that this molecular classification has potential significance in the prognosis assessment of BCa.

### 3.2 Characterization of molecular subtypes

**Figure 3:**
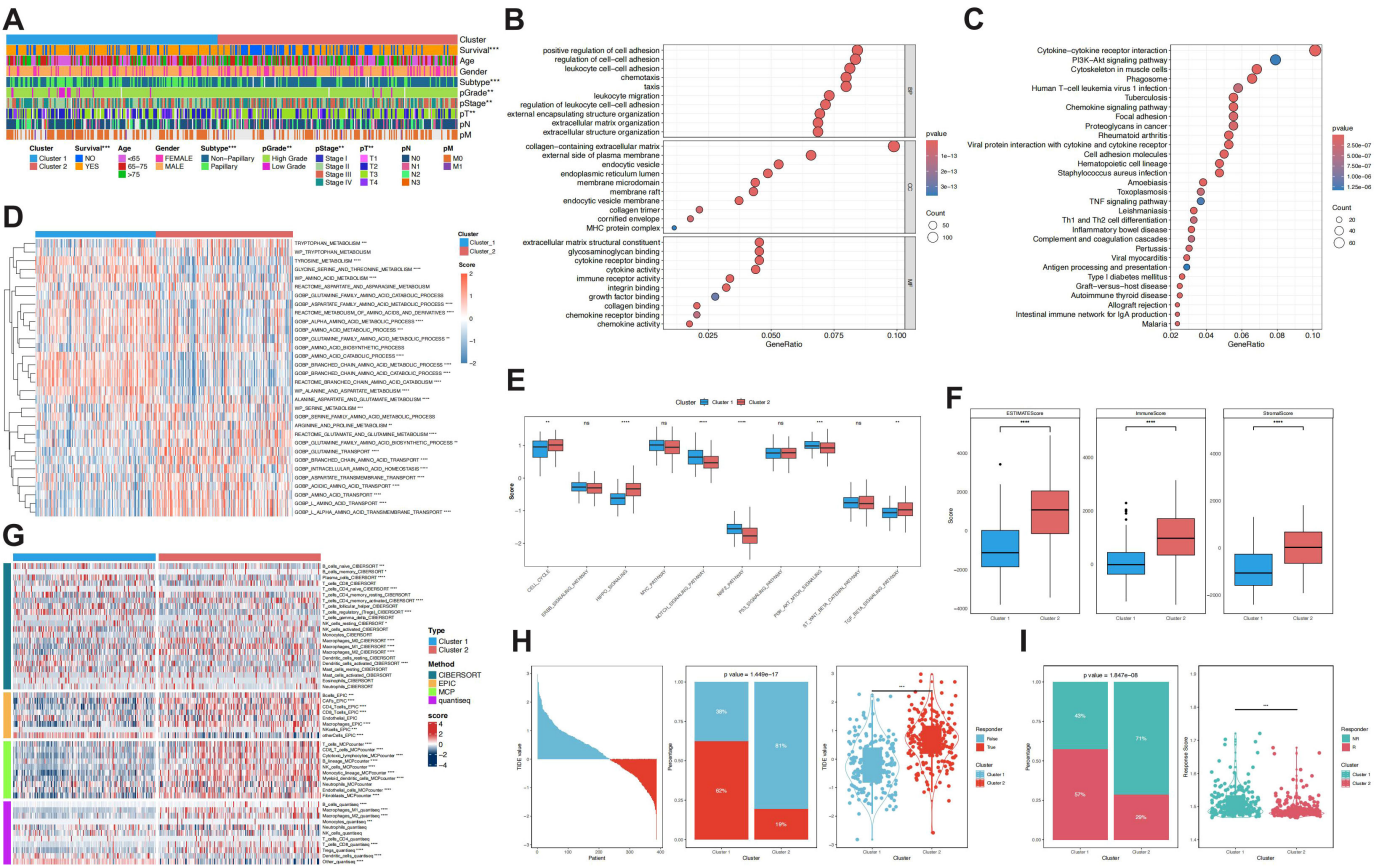
Characteristic differences between molecular subtypes, functional enrichment analysis, immune infiltration differences, and immunotherapy response comparison. **(A)** Comparison of subtypes based on survival, staging, and other clinical features. **(B)** GO enrichment analysis of differentially expressed genes between subtypes. **(C)** KEGG enrichment analysis of differentially expressed genes between subtypes. **(D)** Activity differences of amino acid metabolism gene sets across molecular subtypes as assessed by GSVA. **(E)** Differences in cancer pathway activity across molecular subtypes based on GSVA. **(F)** Immune infiltration analysis based on ESTIMATE. **(G)** Immune cell infiltration level differences evaluated by CIBERSORT, EPIC, MCPcounter, and quantiseq algorithms from the “IOBR” package. **(H)** Immunotherapy response analysis from the TIDE model. **(I)** Immune response analysis from the ImmuneAI algorithm.

Cluster 2 was significantly associated with poor prognostic features, including elevated mortality, predominance of non-papillary histology, higher tumor grade, advanced clinical stage, and increased T classification (all p< 0.01). In contrast, Cluster 1 was more often linked to papillary morphology, early-stage disease, and improved survival, suggesting a less aggressive tumor phenotype (**Figure 3A**). GO enrichment analysis of 1,436 subtype-specific DEGs indicated predominant involvement in pathways regulating cell adhesion, leukocyte trafficking, chemotaxis, exosome organization, and immune receptor interaction (**Figure 3B**). KEGG analysis revealed marked enrichment in immune and inflammatory signaling, including cytokine receptor interactions, chemokine pathways, T-cell differentiation, and antigen presentation, pointing to intensified immune activity and microenvironmental remodeling in Cluster 2 (**Figure 3C**).

At the metabolic level, GSVA revealed subtype-specific amino-acid activities (**Figure 3D**). Cluster 2 was characterized by marked up-regulation of glutamine, tryptophan, branched-chain amino-acid, serine, and tyrosine pathways, implying strengthened transport, assimilation, and reprogramming. Conversely, Cluster 1 preferentially engaged catabolic circuits, including glycine–serine–threonine, tyrosine, and branched-chain amino-acid degradation. Inter-pathway correlations showed that transport-and-biosynthesis routes enriched in Cluster 2 were largely anticorrelated with the core metabolic networks active in Cluster 1, forming a functional axis centred on glutamate, branched-chain amino-acids, and glutamine (**Supplementary Figure 1A**). Hence, Cluster 2 appears driven by metabolic rewiring and adaptive plasticity.

In tumour-associated signalling, NOTCH, NRF2, and PI3K–AKT–mTOR signalling were significantly elevated in Cluster 1 (all p < 0.001). In contrast, Cluster 2 exhibited higher scores in proliferative programmes, including cell-cycle, Hippo, and TGF-β signalling (all p < 0.01). These results suggest that the two clusters may differ in pathway activity, with Cluster 1 tending to be enriched in oxidative stress– and growth-related signalling, whereas Cluster 2 showing a tendency toward stronger activation of proliferation-associated pathways (**Figure 3E**, **Supplementary Figure 1B**).

ESTIMATE analysis indicated significantly greater ImmuneScore, StromalScore, and composite ESTIMATEScore in Cluster 2 (all p< 0.0001; **Figure 3F**). Concordant results from CIBERSORT, EPIC, MCP-counter, and quanTIseq demonstrated enrichment of diverse immune subsets—including CD4^+^/CD8^+^ T cells, Th1/Th17 cells, Tregs, NK cells, macrophages, dendritic cells, MAIT cells, and γδT cells—and a higher overall infiltration score in Cluster 2 (**Figure 3G**, **Supplementary Figures 1C, D**), suggesting an immune-active yet tightly regulated milieu.

TIDE predicted a higher immune-escape score for Cluster 2 (p< 0.001) and a response rate of 19%, markedly below the 62% estimated for Cluster 1 (p< 0.0001). Immune-function signatures (IFNG, CD8, Merck18, PD-L1, CAF, Dysfunction, Exclusion) were likewise increased in Cluster 2, portraying an “activated-yet-suppressed” state (**Figure 3H**, **Supplementary Figure 2**). ImmuneAI further indicated superior response potential for Cluster 1 (57% vs 29%; p< 0.0001) with higher response scores (p< 0.001; **Figure 3I**). Consistently, Cluster 2 displayed elevated scores across multiple immune cell types, including Cytotoxic T cells, Exhausted T cells, and Macrophages, reflecting an activated and diverse immune milieu, whereas Cluster 1 was enriched for CD8_naive and Th17 populations, indicative of a distinct immune profile (**Supplementary Figure 3**). Notably, despite Cluster 2 exhibiting a lower potential for immunotherapy response, its significant immune cell infiltration and activation signals suggest underlying heterogeneity. This necessitates further molecular profiling to elucidate functional phenotypes that may harbor latent therapeutic vulnerabilities associated with immune activation.

### 3.3 Development and validation of a machine learning-based prognostic model

**Figure 4:**
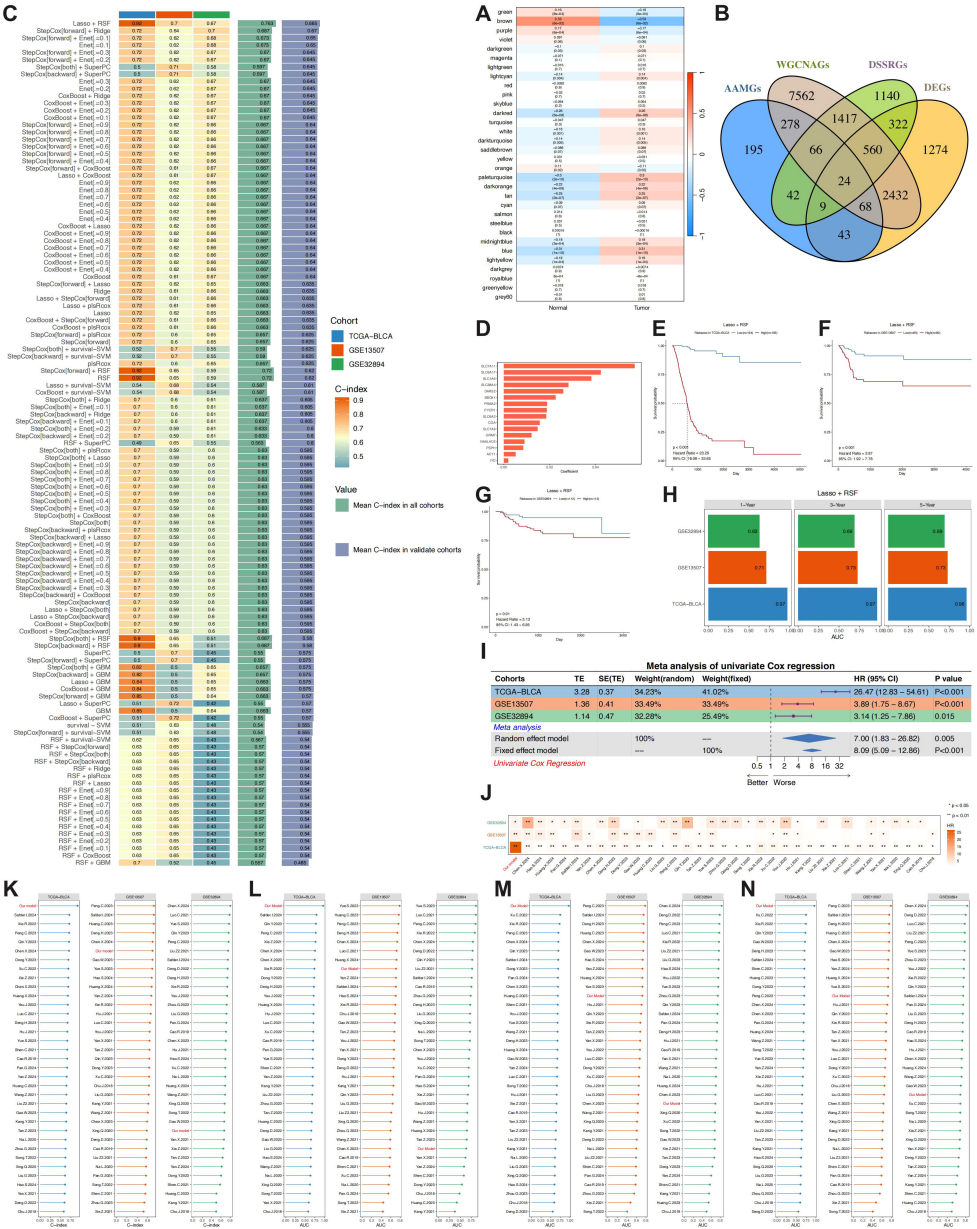
Development of prognostic label based on amino acid metabolism-related genes. **(A)** WGCNA analysis to explore tumor-associated modules. **(B)** Intersection of amino acid metabolism-related genes (AAMRGs), WGCNA genes (WGCNAGs), disease-specific survival-related genes (DSSRGs), and differentially expressed genes (DEGs) to identify candidate genes. **(C)** Identification of optimal algorithm combinations for training (TCGA-BLCA) and validation (GSE13507 and GSE32894) cohorts using 10 machine learning algorithms with 117 combinations with the “mime” package. **(D)** Core genes and their coefficients identified by the best-performing model, LASSO + RSF. **(E-G)** Survival difference analysis between risk groups based on median risk score in TCGA-BLCA **(E)**, GSE13507 **(F)**, and GSE32894 **(G)** cohorts. **(H)** AUC values for 1, 3, and 5 years from the best model across the three cohorts. **(I)** Meta-analysis of univariate regression results from the three cohorts. **(J)** Comparison of univariate regression analysis between the proposed model and previously published models in the training and validation cohorts. **(K)** Comparison of C-index between the proposed model and previously published models. **(L-N)** Comparison of 1, 3, and 5-year AUC values between the proposed model and previously published models.

Univariate Cox regression applied to TCGA tumour samples identified 3–580 genes significantly associated with disease-specific survival (p< 0.05). After excluding outlier specimens and low-abundance transcripts, a weighted gene-co-expression network was constructed with β = 10, yielding 32 modules (**Supplementary Figures 4A–C**). Eleven modules were positively correlated with tumour tissue (p< 0.05 for all; **Figure 4A**), suggesting their potential role in tumourigenesis and progression. Intersection of differentially expressed genes, DSS-related genes, WGCNA modules, and amino-acid-metabolism genes produced 24 candidates (**Figure 4B**, **Supplementary Figure 5**).

Next, we compared 117 machine learning methods to establish a robust prognostic model. The Lasso+RSF combination algorithm yielded the best results, with C-index values of 0.763 in the training set and 0.685 in the validation set (**Figure 4C**). The final prognostic model included 16 key genes (**Figure 4D**), and the corresponding risk score formula was calculated as follows: Risk Score = (0.0570×SLC7A11) + (0.0421×SLC6A17) + (0.0380×SLC1A6) + (0.0282×SLC38A4) + (0.0258×DARS2) + (0.0226×BBOX1) + (0.0189×PSMA2) + (0.0187×PYCR1) + (0.0185×SLC6A2) + (0.0156×CGA) + (0.0147×SLC7A9) + (0.0120×GRM7) + (0.0090×NAALAD2) + (0.0085×PSPH) + (0.0052×ACY1) + (0.0019×IYD). Patients were classified into high and low-risk groups based on the median risk score. Kaplan-Meier survival analysis showed that the high-risk group had significantly worse survival in the TCGA, GSE13507, and GSE32894 cohorts (log-rank P< 0.05) (**Figures 4E–G**). Time-dependent ROC analysis confirmed strong predictive accuracy at 1, 3, and 5 years, with AUC of 0.97, 0.97, and 0.96 in TCGA; 0.71, 0.73, and 0.73 in GSE13507; and 0.63, 0.69, and 0.69 in GSE32894 (**Figure 4H**, **Supplementary Figure 6**).

In three cohorts, univariate Cox analysis confirmed that they all had a HR greater than 1 and P< 0.05. Meta-analysis using both fixed-effect and random-effect models also confirmed that the cohorts was significantly correlated with poor prognosis (HR > 1, p< 0.01) (**Figure 4I**). A comparative analysis with 35 previously published prognostic models revealed that our model outperformed the majority of these models (**Figure 4J**). This superiority was evident across several evaluation metrics, including univariate regression, C-index, and the AUC values for 1-, 3-, and 5-year survival (**Figures 4K–N**).

### 3.4 Clinical features comparison and nomogram construction for prognostic prediction based on risk assessment

**Figure 5:**
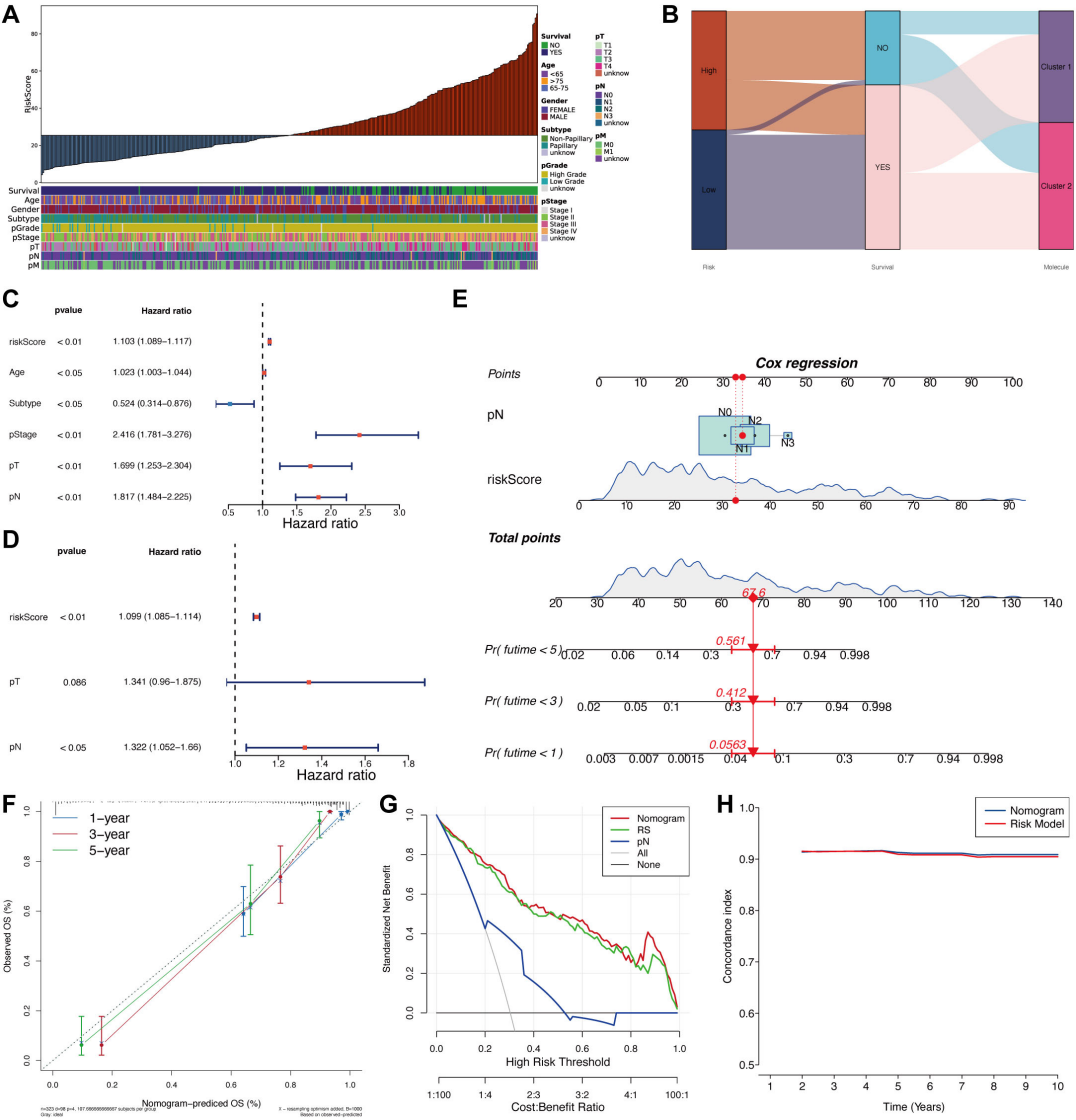
Clinical feature comparison and clinical application potential assessment based on the risk model. **(A)** Distribution of clinical features between high- and low-risk score samples. **(B)** Association of risk groups, survival status, and molecular subtype samples. **(C)** Univariate regression analysis of clinical features, including risk score. **(D)** Multivariate regression analysis of significant features to identify independent prognostic factors. **(E)** Construction of a nomogram based on multivariate regression significant results. **(F)** Calibration curve to evaluate the stability of 1-, 3-, and 5-year predictions. **(G)** Decision curve analysis comparing the net benefit of different clinical strategies. **(H)** C-index analysis of the nomogram and risk model.

The risk score exhibited significant associations with various clinical and pathological features. Patients in the high-risk group were more likely to experience poor survival outcomes and showed worse characteristics in histological subtypes, pathological grade, clinical stage, tumor infiltration depth, lymph node metastasis, and distant metastasis. These features were predominantly linked to non-papillary subtypes, high-grade tumors, advanced stages (Stage III–IV), T2–T4 stages, N1–N3 stages, and M1 stage (**Figure 5A**, **Supplementary Figures 7, 8**). Sankey diagrams illustrated the survival status and molecular subtype distribution within the risk groups (**Figure 5B**). The risk score captured most death events and was strongly associated with adverse outcomes. Notably, these events were enriched in the poor-prognosis subtype (Cluster 2), whereas high-risk patients were distributed across both clusters, suggesting that the risk score offers prognostic information complementary to molecular subtyping.

Cox proportional hazards regression identified the risk score as a statistically independent prognostic factor. In univariate analysis (**Figure 5C**), significant associations with disease-specific survival were observed for the risk score, age, molecular subtype, pathological stage (pStage), tumor invasion depth (pT), and lymph node status (pN) (p< 0.05). Multivariate analysis incorporating all significant variables (**Figure 5D**) confirmed the independent prognostic value of the risk score (HR = 1.099, 95% CI: 1.068–1.131, p< 0.01). Although pT displayed a potential risk trend (p = 0.066), statistical significance was retained only for pN (HR = 1.322, p< 0.05).

A nomogram incorporating the risk score and pN was constructed to predict individualized survival probabilities at 1, 3, and 5 years (**Figure 5E**). Total points were calculated by summing the contributions of each variable, and survival likelihood was projected along a reference axis. Calibration curves demonstrated close agreement between predicted and actual outcomes across all time points, indicating strong predictive accuracy (**Figure 5F**). Decision curve analysis showed that the nomogram offered greater net clinical benefit across a wide range of threshold probabilities compared to single-variable or null models, underscoring its practical utility (**Figure 5G**). Consistently high C-index values (>0.9) were observed throughout the 10-year follow-up, with minimal variation, suggesting that both the nomogram and the original risk score model maintain stable and reliable long-term predictive performance (**Figure 5H**).

### 3.5 Pathways and drug sensitivity associated with the risk model

**Figure 6:**
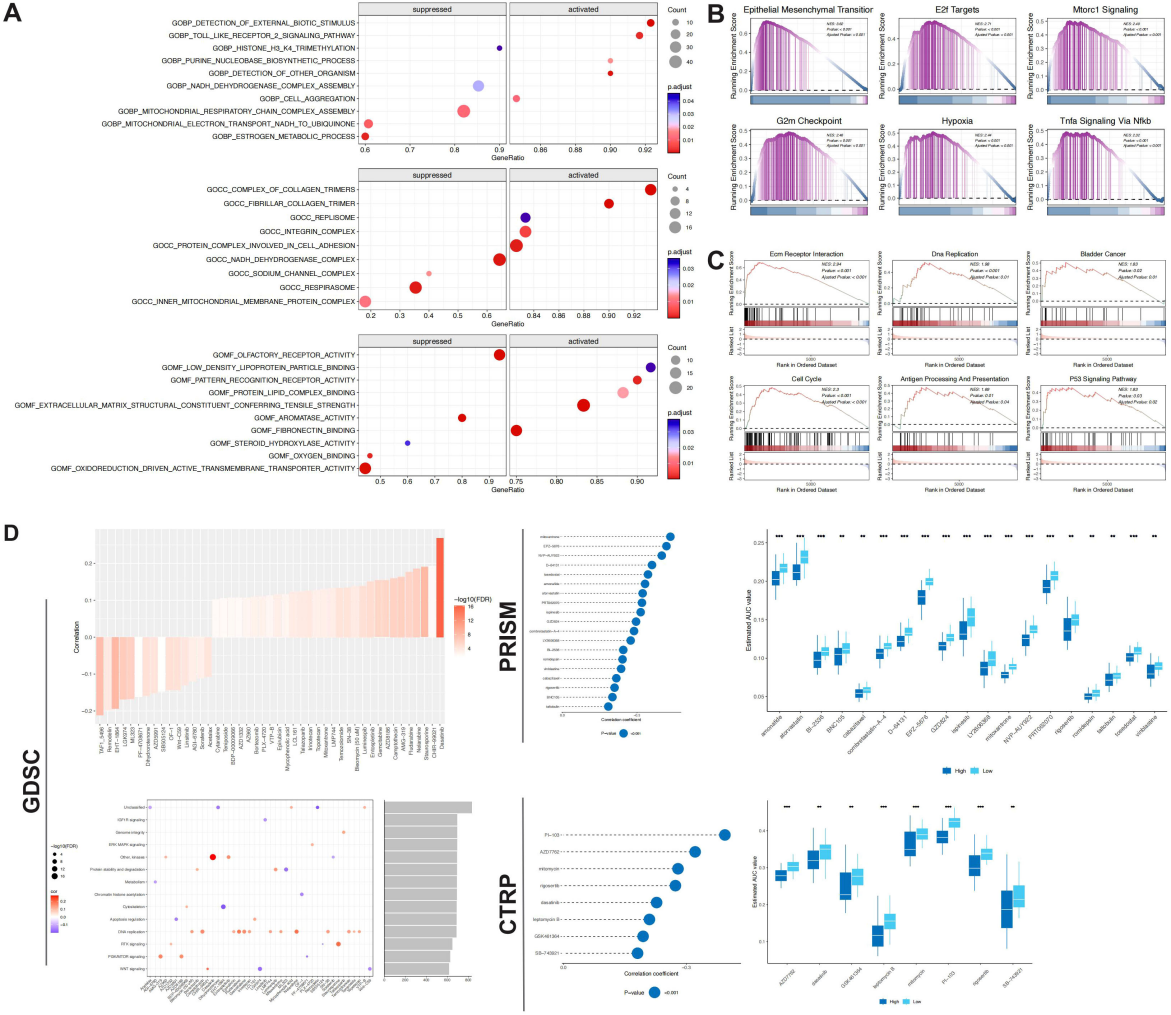
Pathway activity assessment and potential drug differences between risk groups. **(A-C)** GSEA analysis of GO, KEGG, and HALLMARK gene set enrichment differences between risk groups. **(D)** GDSC data showing drugs and pathways associated with the prognostic model. **(E)** PRISM and CTRP data identifying potential drugs that may benefit high-risk groups.

Functional enrichment analysis revealed significant activation of multiple proliferation and stress-related pathways in the high-risk group (**Figures 6A–C**). Hallmark pathways enriched included the cell cycle, E2F activation, mTORC1 signaling, hypoxia response, and TNF-αmediated inflammation. GO analysis highlighted upregulation of processes related to pathogen recognition, Toll-like receptor signaling, extracellular matrix structure, and adhesion, while mitochondrial metabolism, steroid synthesis, and electron transport were downregulated, indicating metabolic reprogramming. KEGG analysis further indicated active involvement in DNA replication, cell cycle regulation, p53 signaling, antigen processing, and matrix-receptor interactions, suggesting a stronger proliferative capacity, immune response, and tissue remodeling potential in the high-risk group.

Drug sensitivity predictions revealed therapeutic differences in the risk groups (**Figure 6D**). In the GDSC database, several drugs, such as tyrosine kinase inhibitors (Dasatinib, Staurosporine), WNT pathway regulators (CHIR-99021), PI3K/mTOR inhibitors (AMG-319, AZD8186), DNA-damaging agents (Fludarabine, Gemcitabine, SN-38, Topotecan, Temozolomide), and proteostasis regulators (Bortezomib, Luminespib), showed positive correlation with the risk score, targeting pathways like RTK, ERK-MAPK, PI3K/AKT/mTOR, DNA replication, and cytoskeleton dynamics, aligning with the active biological state in the high-risk group. In contrast, drugs negatively correlated with the risk score, such as Remodelin, LGK974, AZD5991, PF-4708671, and Linsitinib, mainly targeted the WNT signaling, IGF1R axis, metabolic stability, and chromatin modification pathways, potentially benefiting the low-risk group.

Further validation from the PRISM and CTRP databases highlighted potential therapies with preferential efficacy in high-risk patients (**Figure 6D**). In PRISM, drugs like Mitoxantrone, Romidepsin, Cabazitaxel, and Taltobulin showed lower AUC values in the high-risk group, involving DNA topology, epigenetics, and microtubule disruption mechanisms. Similarly, in CTRP, PI-103, AZD7762, Mitomycin, Rigosertib, and Dasatinib exhibited stronger sensitivity in the high-risk group, targeting PI3K signaling, cell cycle inhibition, and multi-target kinase pathways.

### 3.6 Immune infiltration and immunotherapy response stratified by prognostic model

**Figure 7:**
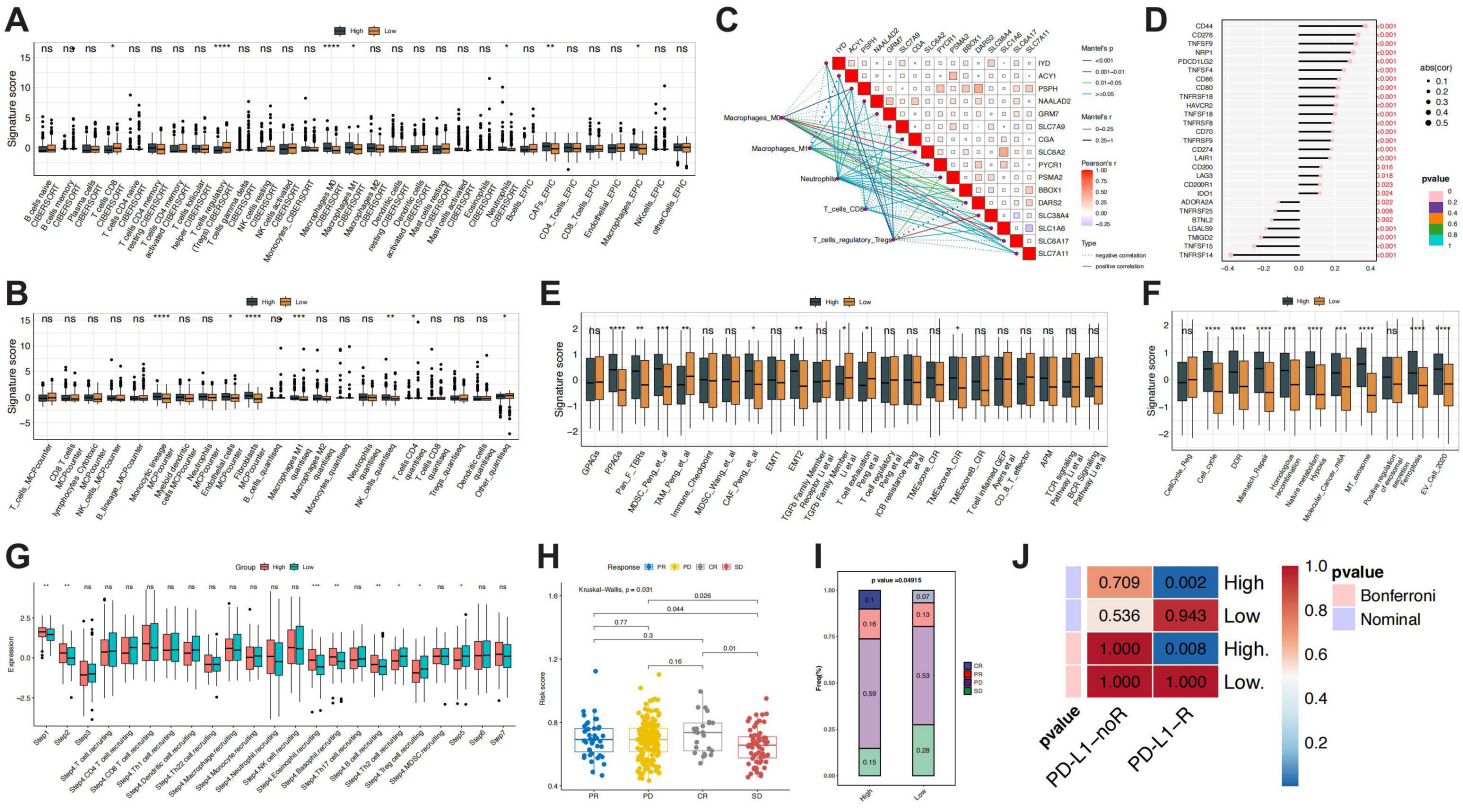
Immune landscape differences between risk groups. **(A)** Evaluation of immune cell infiltration levels between high- and low-risk groups using the CIBERSORT and EPIC algorithms. **(B)** Evaluation of immune cell infiltration levels between high- and low-risk groups using the MCPcounter and quantiseq algorithms. **(C)** Correlation between differentially infiltrated immune cells (based on CIBERSORT results) and the prognostic model. **(D)** Correlation between risk scores and immune checkpoint markers. **(E, F)** Differences in immune-related signatures between risk groups. **(G)** Potential immune therapy response differences between risk groups, based on TIP immune therapy response evaluations. **(H, I)** Application of the prognostic model in the Imvigor 210 immunotherapy cohort. **(J)** Identification of potential PD-L1 responder groups using the Submap algorithm. * p < 0.05, ** p < 0.01, *** p < 0.001, **** p < 0.0001.

Distinct differences in the immune microenvironment were observed between the risk groups. CIBERSORT analysis revealed that the high-risk group had significantly upregulated M0 macrophages, M1 macrophages, and neutrophils, while the low-risk group was predominantly enriched with CD8^+^ T cells and Tregs. Further support came from the EPIC and MCP-counter algorithms, which showed increased infiltration of cancer-associated fibroblasts (CAFs), macrophages, monocytic lineage cells, fibroblasts, and endothelial cells in the high-risk group. Quantiseq analysis indicated a higher proportion of M1 macrophages and NK cells in the high-risk group, while CD4^+^ T cells and unclassified cells were more abundant in the low-risk group (**Figures 7A, B**). These findings suggest that the immune status of the high-risk group is skewed toward immune-activated inflammation, while the low-risk group reflects moderate immune clearance.

Immunological correlation analysis further revealed specific interactions between model genes and key immune cells. Mantel test results showed positive correlations between Tregs and genes such as *ACY1*, *IYD*, and *PSMA2*, while negative correlations were found with *BBOX1*, *DARS2*, and *PSPH*. Macrophages (M0/M1) exhibited bidirectional relationships with metabolism- and stress-related genes. CD8^+^ T cells demonstrated negative correlations with *SLC1A6* and *SLC6A2* (**Figure 7C**). Correlation analysis also identified stable co-expression patterns among several model genes, forming a robust functional synergy network. Additionally, the high-risk group exhibited upregulation of immune co-stimulatory molecules (*CD44*, *CD276*) and the co-inhibitory molecule *PDCD1LG2*, consistent with higher scores in immune-related signatures and biological pathways involved in proliferation, stress adaptation, and metabolic reprogramming, including myeloid-derived suppressor cells, pan-cancer proliferation-associated genes, ferroptosis, and Molecular cancer-associated N6-methyladenosine regulatory signature (**Figures 7D–F**). TIP analysis further indicated enhanced immune activation, granulocyte, and B cell recruitment in the early stages of immune response in the high-risk group. In contrast, the low-risk group exhibited higher scores in Treg and Th2 cell recruitment, as well as later immune effect stages, highlighting immune regulation and activation heterogeneity between the groups (**Figure 7G**).

Immunotherapy response analysis revealed that patients in the high-risk group had higher overall risk scores in the Imvigor210 immunotherapy cohort, with significantly higher scores across CR, PR, and PD subtypes compared to SD (p = 0.031) (**Figure 7H**). Immune response composition analysis showed that 59% of the high-risk group exhibited PD, with CR and PR responses collectively accounting for 26%, and a relatively low SD rate (15%). In contrast, the low-risk group had 20% CR and PR responses, with SD increasing to 28% and PD at 53%. The differences in response composition between the groups were statistically significant (p = 0.04915) (**Figure 7I**). SubMap analysis further confirmed that the high-risk group was significantly enriched in the PD-L1 treatment response group (Nominal p = 0.002, Bonferroni p = 0.008) (**Figure 7J**), suggesting that the risk scoring system may identify patients likely to benefit from immunotherapy.

### 3.7 Single-cell analysis and screening of key candidate genes

**Figure 8:**
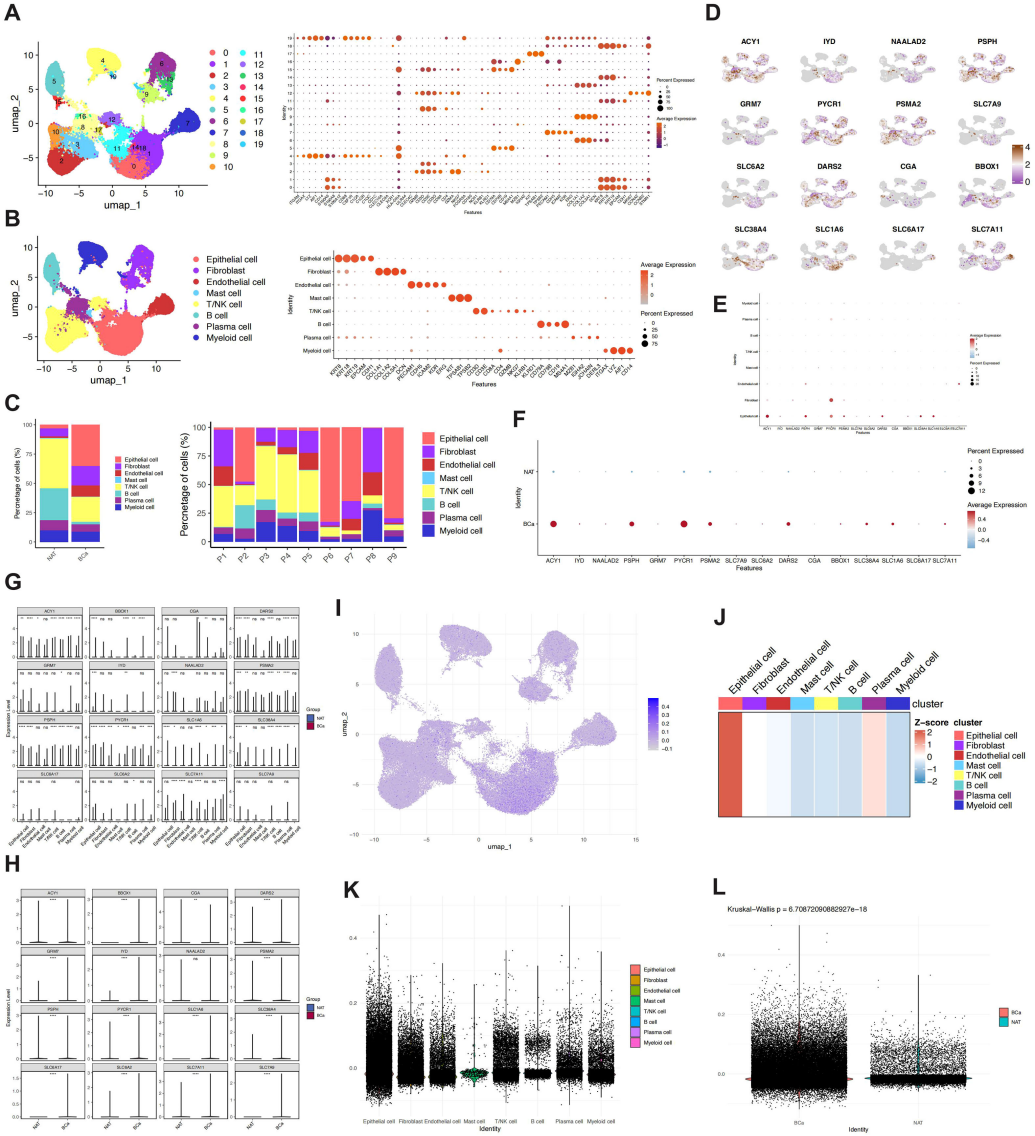
Expression and activity scoring of model genes in single-cell data. **(A)** UMAP showing data annotation into 19 categories based on marker genes. **(B)** UMAP displaying cell annotation information across eight cell types. **(C)** Cell composition proportions across different groups and samples. **(D-H)** Expression differences of genes across different cell types and groups. **(I-L)** Activity differences of the model gene set between cells and groups.

Single-cell RNA sequencing data were processed through quality control, normalization, and high-variance gene selection. Initial dimensionality reduction was performed using PCA, followed by visualization via UMAP (**Supplementary Figure 9**). Clustering was conducted using the Louvain algorithm at a resolution of 0.5, identifying 19 distinct cell clusters (**Figure 8A**). These clusters were annotated into eight major cell types based on canonical marker gene expression (**Figure 8B**), including epithelial cells, fibroblasts, endothelial cells, mast cells, T/NK cells, B cells, plasma cells, and myeloid cells. Representative markers—such as *KRT8* and *EPCAM* for epithelial cells, *PECAM1* and *CDH5* for endothelial cells, and *S100A8*, *LYZ*, and *ITGAX* for myeloid cells—validated the accuracy of annotation.

Comparative analysis between tumor tissue (BCa) and adjacent non-tumor tissue (NAT) revealed higher proportions of epithelial cells, fibroblasts, and endothelial cells in BCa, whereas T/NK and B cells were more enriched in NAT (**Figure 8C**). The 16 genes from the prognostic model were predominantly expressed in epithelial cells, with overall higher expression in BCa than in NAT (**Figures 8D–H**). These genes were concentrated in epithelial clusters enriched within tumor regions, suggesting their involvement in tumor-associated epithelial phenotypes. These cells likely represent malignant epithelial populations or cells in an activated state. A custom signature score constructed from the 16-gene set was significantly elevated in epithelial clusters within UMAP space (**Figures 8I, J**), with higher scores in epithelial cells compared to other lineages (**Figure 8K**) and significantly higher in BCa than in NAT (p< 0.0001) (**Figure 8L**), supporting the role of this gene set in tumor-related epithelial activity.

Prioritized genes associated with tumor biology were identified through systematic screening of the TCGA cohort. Differential expression was analyzed in both unpaired and paired tumor-adjacent samples, alongside subtype-specific comparisons and survival analysis (**Supplementary Figures 10, 11**). Integrative filtering yielded three candidates—*DARS2*, *PSPH*, and *SLC6A17*—which were consistently upregulated in tumors, differed across molecular subtypes, and were significantly linked to poor prognosis, warranting further investigation.

### 3.8 Gene expression validation in tissue and cell lines

**Figure 9:**
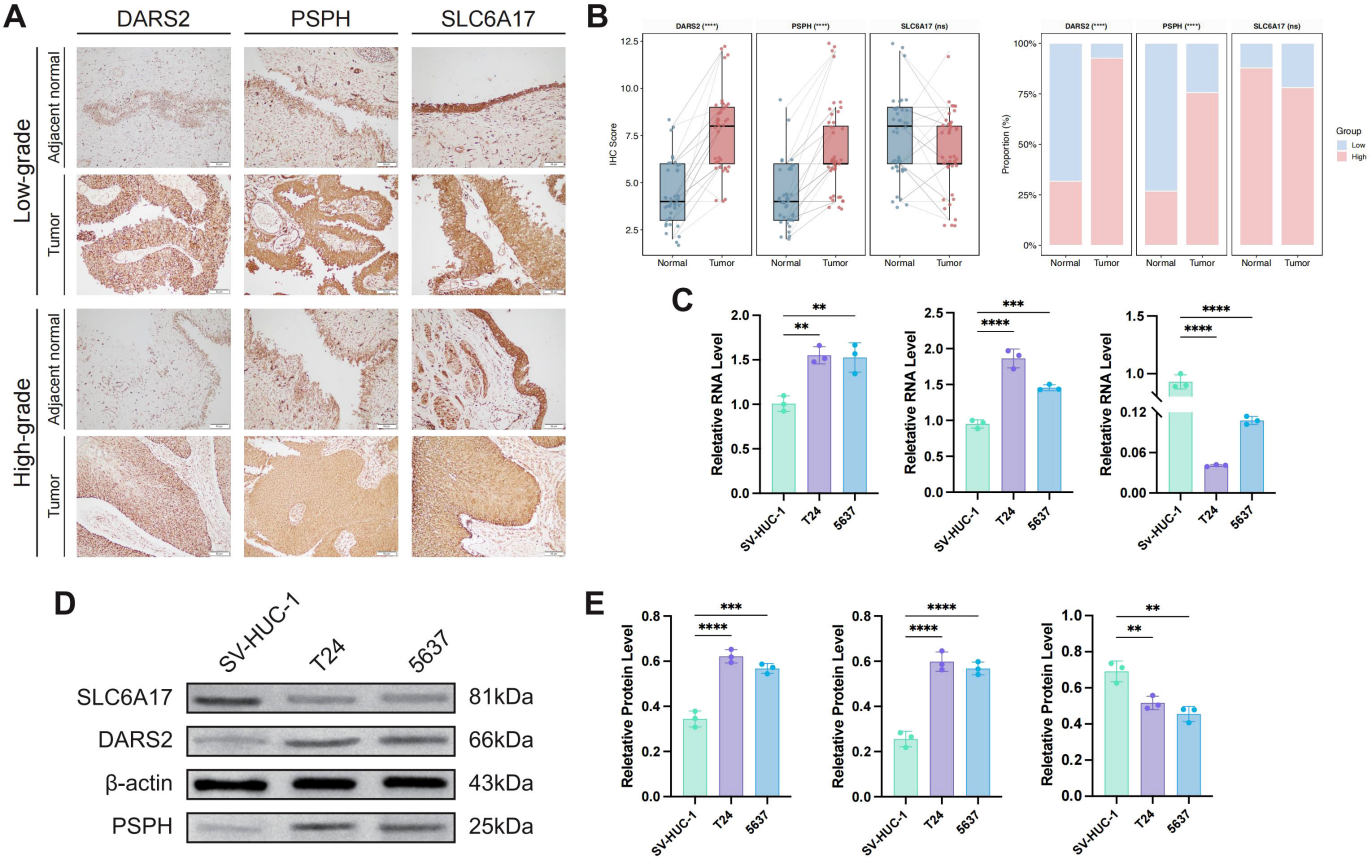
Gene expression analysis of DARS2, PSPH, and SLC6A17 in bladder cancer tissues and cell lines. **(A)** Immunohistochemical staining of DARS2, PSPH, and SLC6A17 in high-grade (n=34) and low-grade (n=7) bladder urothelial carcinoma tissues. **(B)** Paired tests of continuous and categorical expression data for the three genes, displayed as boxplots and bar charts, highlighting significant differences between tumor and normal tissues. **(C)** Expression of *DARS2*, *PSPH*, and *SLC6A17* in SV-HUC-1 normal cells and T24, 5637 tumor cell lines. **(D, E)** Western blot analysis **(D)** showing protein expression levels of DARS2, PSPH, and SLC6A17 in T24 and 5637 cells, with statistical differences **(E)** compared to SV-HUC-1 cells.

Immunohistochemical analysis of tumor and adjacent normal tissues from 41 patients showed that DARS2, PSPH, and SLC6A17 are primarily cytoplasmic. Both DARS2 and PSPH exhibited significantly higher expression in tumor tissues than in normal tissues (all p< 0.05), whereas SLC6A17 did not show significant differences (**Figures 9A, B**). Consistent trends were observed in high-grade (n=34) and low-grade (n=7) urothelial carcinoma samples (**Supplementary Figures 12A, B**). Correlation with TCGA clinical data revealed significant associations between these gene expressions and patient survival, as well as several clinical characteristics (**Supplementary Figure 13**).

In cell line experiments, *DARS2* and *PSPH* were upregulated in T24 and 5637 BCa cells compared to the SV-HUC-1 control (all p< 0.05), while *SLC6A17* showed reduced expression (**Figure 9C**). These findings were confirmed by Western blot analysis, which showed elevated DARS2 and PSPH levels in cancer cell lines, with SLC6A17 being downregulated (**Figures 9D, E**). Based on these consistent expression patterns and prior research on *DARS2* in BCa, *PSPH* was selected for further investigation (38, 39).

### 3.9 Functional validation of PSPH *in vitro*

**Figure 10:**
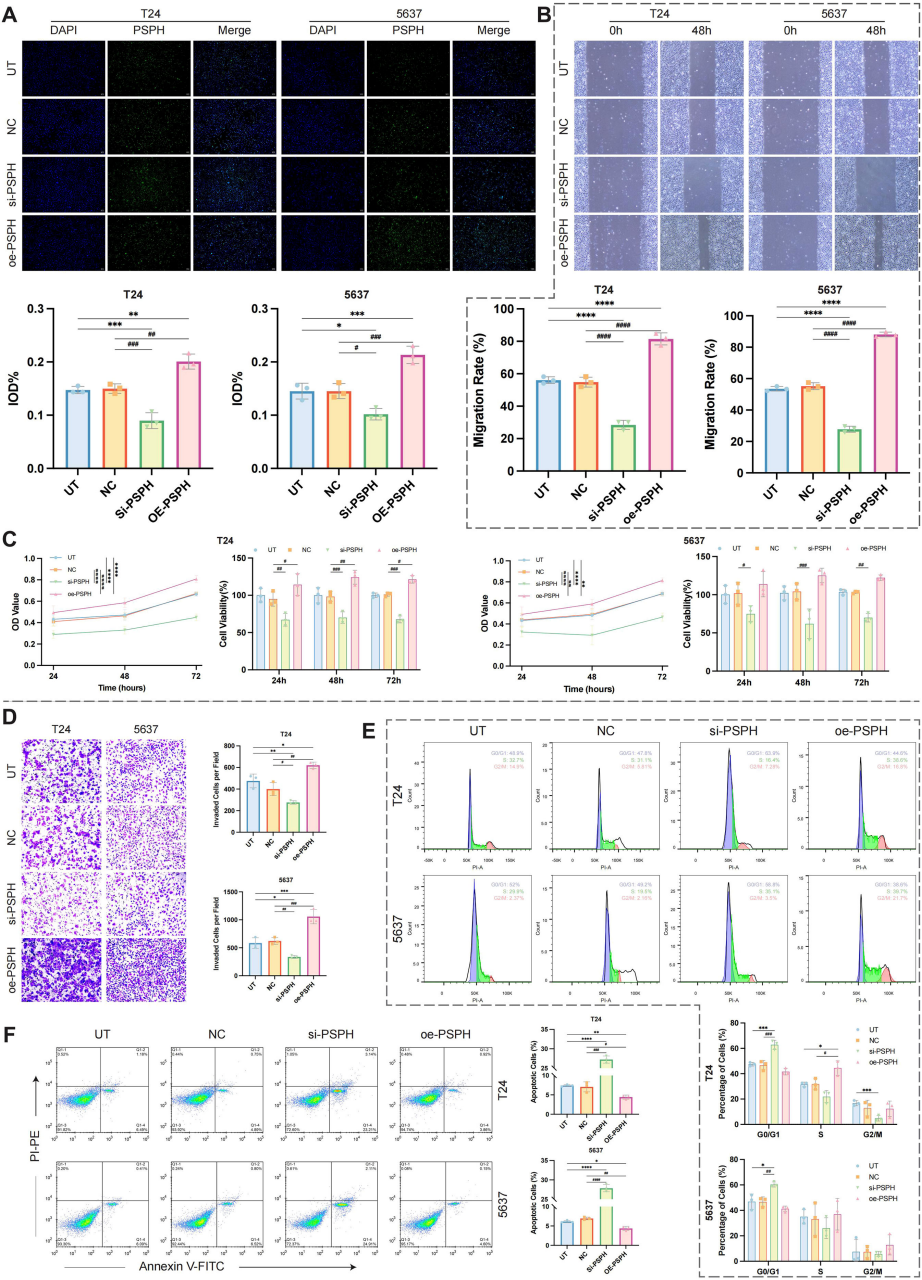
Functional validation of *PSPH* in T24 and 5637 bladder cancer cell lines. **(A)** Immunofluorescence staining of *PSPH* in T24 and 5637 cells, showing expression in untreated (UT), negative control (NC), knockdown (si-PSPH), and overexpression (oe-PSPH) groups. *PSPH* expression was quantified by measuring Integrated Optical Density (IOD), representing the fluorescence intensity of each group. **(B)** Wound-healing assay at 48 hours, with representative images of cell migration and statistical analysis of migration rates across groups. **(C)** CCK-8 assay showing OD values at 24, 48, and 72 hours for T24 and 5637 cells in all four experimental groups, along with statistical analysis of cell viability. **(D)** Transwell invasion assay images and statistical analysis at 48 hours for T24 and 5637 cells in all four experimental groups. **(E)** Flow cytometry analysis of cell cycle distribution at 48 hours for T24 and 5637 cells, showing the proportions of cells in G0/G1, S, and G2/M phases, with corresponding statistical analysis. **(F)** Flow cytometry analysis of apoptosis at 48 hours in T24 and 5637 cells, with trend graphs and statistical analysis of apoptotic rates across all four groups. For comparisons versus the UT group: * p < 0.05, ** p < 0.01, *** p < 0.001, **** p < 0.0001. For comparisons versus the NC group: # p < 0.05, ## p < 0.01, ### p < 0.001, #### p < 0.0001.

*PSPH* overexpression plasmids and siRNA constructs were introduced into the T24 cell line to assess their functional effects. The si-670 construct effectively knocked down *PSPH* expression, while the overexpression plasmid significantly upregulated *PSPH* levels compared to the untreated (UT) and empty vector (NC) controls (p< 0.05) (**Supplementary Figures 14A–D**). Immunofluorescence staining revealed that PSPH localized primarily in the cytoplasm, with significant expression differences observed between the knockdown, overexpression, and control groups (p< 0.05) (**Figure 10A**, **Supplementary Figure 14E**).

Functional assays confirmed the impact of *PSPH* on cellular behavior. Wound-healing assays showed decreased migration in the knockdown group and increased migration in the overexpression group after 48 hours (**Figure 10B**). CCK-8 assays indicated significant differences in optical density at 24, 48, and 72 hours between experimental and control groups (p< 0.05). Cell viability analysis demonstrated significant differences between T24 knockdown and overexpression groups, and the knockdown group in 5637 cells also differed significantly from NC (**Figure 10C**). Transwell invasion assays showed increased invasion in the overexpression group and decreased invasion in the knockdown group (p< 0.05) (**Figure 10D**). Flow cytometry analysis of the cell cycle revealed a higher proportion of cells in G0/G1 in both the knockdown groups of T24 and 5637 cells, while overexpression in T24 cells increased the proportion of cells in the S phase. In T24 cells, the knockdown group also showed a significantly lower proportion of cells in G2/M (p< 0.05) (**Figure 10E**). Apoptosis assays revealed a significant increase in apoptosis in the knockdown group and a decrease in the overexpression group compared to UT and NC (p< 0.05) (**Figure 10F**).

## 4 Discussion

In this study, by integrating multiple cohorts, we systematically delineated the expression landscape of AAMGs in BCa and identified two molecular subtypes with distinct prognostic, metabolic, and immune features. On this basis, we developed a stable and clinically transferable AAMG-based prognostic model. Single-cell transcriptomic analysis demonstrated that the model genes were predominantly expressed in malignant epithelial cells, indicating that the signature captures intrinsic, metabolism-driven properties of tumor cells rather than passive admixture of microenvironmental components. Functional assays further established *PSPH* as a key oncogenic driver within this network, underscoring the importance of amino acid metabolic reprogramming in shaping the malignant phenotype of BCa.

The two AAMG-defined subtypes primarily align closely with the increasingly recognized concept of “metabolism–immune coupling” in BCa. Cluster 2 was characterized by upregulation of glutamine, branched-chain amino acid, tryptophan, and serine “uptake–biosynthesis” pathways, accompanied by poor prognosis and an immunosuppressive phenotype, mirroring previously described glutamine-dependent high-risk groups (24). Prior studies have shown that metabolic reprogramming can directly dictate the functional states of tumor and immune cells. For instance, the glutamine antagonist JHU-083 disrupts metabolic homeostasis in bladder and prostate cancer models and reprograms TAMs toward a pro-inflammatory phenotype, thereby enhancing CD8^+^ cytotoxicity (40). Conversely, in renal cancer, glutamine deprivation triggers EGFR–ERK–c-Jun activation and PD-L1 upregulation, suggesting that single-pathway inhibition may evoke compensatory immunosuppressive feedback (41). Our subtypes also recapitulate the “immune-hot but dysfunctional” pattern: although Cluster 2 displayed abundant immune infiltration and higher ESTIMATE scores, it simultaneously exhibited increased TIDE scores and reduced predicted immunotherapy responsiveness, indicating a disconnection between infiltration and effector function. Consistent with previous work, aberrant amino acid metabolism can impair CD8^+^ T-cell persistence through nutrient competition (e.g., glutamine uptake), immunosuppressive metabolites (e.g., the Kyn–AhR axis), and arginine depletion or NO-mediated T-cell inhibition, collectively producing an “infiltrated yet ineffective” immune landscape (42–50). These findings suggest that the AAMG-based metabolic subtyping captures, at least in part, coupled metabolic–immune features underlying immunological heterogeneity in BCa. Meanwhile, accumulating evidence indicates that RNA-based regulation beyond protein-coding programs also contributes to malignant progression and stemness. For instance, ADAMTS9-AS1 has been implicated in promoting BCa progression, whereas YAP1/YTHDF3/SMAD7 signaling has been linked to stemness regulation (51, 52). Such studies highlight an additional regulatory layer involving non-coding RNA and RNA-modification–associated programs that could be incorporated in future investigations to further elucidate the molecular basis of the metabolism–disease link and its contribution to tumor progression.

The AAMG-based prognostic model demonstrated robust predictive performance across multiple independent cohorts, indicating that the metabolic heterogeneity reflected by the signature not only complements conventional pathological parameters but also carries independent prognostic value. Compared with previous multigene models, our pathway-anchored approach enhances biological interpretability and facilitates integration with emerging metabolism-targeted therapeutic strategies (24, 25, 53). Single-cell analyses further confirmed that the model genes originate primarily from malignant epithelial cells, suggesting that the risk score reflects intrinsic metabolic–signaling states rather than microenvironment-derived transcriptional noise. Mechanistically, the high-risk group showed marked enrichment of cell-cycle progression, DNA replication, mTORC1 activation, and inflammatory stress pathways—hallmarks of metabolically reprogrammed, highly proliferative tumors (54, 55). These features imply potential susceptibility to DDR-targeting agents or PI3K/mTOR inhibitors, whereas low-risk tumors may be more amenable to metabolic or epigenetic therapies. Notably, despite poorer prognosis and stronger proliferative pressure, the high-risk group exhibited higher response rates in immunotherapy cohorts, consistent with its predominant distribution within the immune-competent Cluster 1 (36). As most high-risk cases mapped to Cluster 1, the risk score may further refine molecular subtyping and delineate a subset with adverse outcomes for which the relationship with immunotherapy response deserves further investigation. Together with prior evidence that highly proliferative tumors often display increased neoantigen load and IFN-γ–driven T-cell inflammation—key determinants of anti-PD-1 benefit—our findings suggest that the high-risk group represents a “high-proliferation with T-cell–inflamed” phenotype rather than an immune-exhausted state (56–58). Thus, the metabolic risk score not only stratifies patients with unfavorable outcomes but also identifies subsets likely to derive clinical benefit from immunotherapy, providing a feasible framework for metabolism-informed immunotherapeutic stratification. To assess the clinical utility of the risk score, a nomogram was developed integrating the score and lymph node status for personalized survival predictions. However, additional validation is necessary to confirm its robustness, including testing the proportional hazards assumption and addressing multicollinearity among clinical covariates for broader applicability.

Among the candidate genes, *PSPH* seems to stand out for its distinct expression pattern and functional relevance, and our cellular experiments validated this model-derived signal as a potential oncogenic driver. Extensive evidence across cancer types supports its pleiotropic role in metabolic reprogramming, oxidative stress adaptation, signaling activation, and immune modulation. As the terminal enzyme of the serine synthesis pathway, *PSPH* enhances *de novo* L-serine production and boosts NADPH/GSH availability, enabling tumors such as glioblastoma and hepatocellular carcinoma to sustain growth under nutrient scarcity or elevated ROS (59, 60). In non-small cell lung cancer and esophageal squamous carcinoma, *PSPH* activates MAPK/JNK signaling to promote migration and invasion, whereas in cutaneous squamous carcinoma its oncogenic effects can occur independently of canonical serine synthesis (61–63). Studies in colorectal cancer and hepatocellular carcinoma further show that *PSPH* modulates autophagy and fosters an immunosuppressive microenvironment by upregulating PD-L1, enriching M2-like TAMs, and facilitating immune evasion (60, 64). These cross-cancer findings strongly support the prometastatic and pro-proliferative phenotypes we observed in BCa cells following *PSPH* manipulation. Notably, the work by Ma et al. provides additional mechanistic insight, demonstrating that *PSPH* is regulated by *BACH1* and promotes malignant progression and gemcitabine resistance through the S100A2–MAPK axis (65). These insights are largely consistent with our experimental observations and suggest that *PSPH* may play a key role within our model. While *PSPH* appears to show promise as a high-weight component of the AAMG signature and as a potential therapeutic target, further *in vivo* studies, metabolic flux analysis, and rescue experiments are necessary to fully validate its biological role and therapeutic relevance in BCa.

This study has several limitations. The molecular subtypes and prognostic model were primarily derived from public datasets and require further validation in larger prospective cohorts. Although functional studies on *PSPH* have provided valuable insights, they were mainly conducted *in vitro*, which may not fully capture the metabolic and immune complexities of *in vivo* conditions. Future research could benefit from exploring liquid–liquid phase separation models to investigate amino acid metabolism under gene interventions, as well as examining metabolic changes with material supplementation to improve our understanding of metabolic-immune interactions. Furthermore, *PSPH* may serve as a potential key metabolic node, but the roles of other signature genes remain less defined and warrant further exploration.

## 5 Conclusion

In conclusion, the molecular subtypes and prognostic model derived from amino acid metabolism–related genes reveal distinct differences in functional, immune, therapeutic, and prognostic characteristics of BCa, while suggesting *PSPH* as a key driver with significant biological and clinical implications.

## Data Availability

The original contributions presented in the study are included in the article/**Supplementary Material**. Further inquiries can be directed to the corresponding author.
