# Supplementary material for: A systematic characterization of amino acid metabolism–related genes reveals molecular subtypes and a prognostic signature in bladder cancer
Source: Front Oncol. 2026 Feb 27;16:1754912. doi: 10.3389/fonc.2026.1754912 (PMC12982074; doi:10.3389/fonc.2026.1754912)
Supplement: Supplementary file 1 [file DataSheet1.docx]

Supplementary Material

# Supplementary Methods

1. Immunohistochemistry (IHC)

FFPE bladder cancer tissues and matched adjacent tissues collected between February 2017 and February 2022 were obtained from patients undergoing radical cystectomy. All specimens were acquired in accordance with institutional regulations and approved by the Ethics Committee of Nanchong Central Hospital.

Sections were cut at 4 μm, deparaffinized in xylene, and rehydrated through graded ethanol. Heat-induced antigen retrieval was performed in EDTA buffer using full-pressure steaming for 3 min. Endogenous peroxidase activity was quenched with 3% hydrogen peroxide for 10 min. Primary antibody incubation was conducted using anti-DARS2 (Proteintech, 13807-1-AP; 1:400, 1 h), anti-PSPH (Proteintech, 14513-1-AP; 1:400, 1 h), or anti-SLC6A17 (Affinity, DF9727; 1:600, overnight at 4 °C). After PBS rinses, sections were incubated with an HRP-conjugated secondary antibody raised against the corresponding host species for 30 min and developed using DAB. Brown cytoplasmic staining was interpreted as positive.

Staining intensity (0–3) and the percentage of positive tumor cells (1–4) were assessed, and the two measures were combined to generate a final score. Scores ≤4 were classified as low expression and >4 as high expression. All slides were independently evaluated by two senior pathologists blinded to clinical information; cases with discrepant classifications were reviewed jointly to reach consensus.

Paired continuous variables were analyzed using the Wilcoxon signed-rank test, and paired categorical variables were examined using McNemar’s test. Statistical significance was defined as a two-sided P < 0.05.

1. Cell-Based Experiments
2. Cell Lines and Cell Culture

The human bladder cancer cell lines T24 (CL-0227) and 5637 (CL-0002), and the normal urothelial cell line SV-HUC-1 (CL-0222) were obtained from Procell Life Science & Technology. All cell lines were authenticated by short tandem repeat (STR) analysis and used within limited passages after receipt. T24 cells were cultured in Macrocy’s 5A medium (CM-0227), 5637 cells in RPMI-1640 medium (CM-0002), and SV-HUC-1 cells in Ham’s F-12K medium (CM-0222), each supplemented with 10% fetal bovine serum and 1% penicillin–streptomycin. Cells were maintained at 37 °C in a humidified atmosphere containing 5% CO₂ and passaged using trypsin–EDTA solution (allBio, 03.13005A).

1. RNA Extraction and Quantitative PCR (qPCR)

Total RNA was extracted from cultured cells using TRIzol reagent (Aidlab, RN0102) following the manufacturer’s instructions. Briefly, cells were lysed in TRIzol, followed by phase separation with chloroform, RNA precipitation with isopropanol (Macklin, I811925), washing with 75% ethanol (Macklin, E809056), and dissolution in RNase-free water (DEPC-treated; MDL, MD911875). RNA integrity was verified by agarose gel electrophoresis (UltraPure Agarose, Biomiga, SH441-01), and RNA concentration and purity were determined using a spectrophotometer (Youmi, Unano-1000). Samples with A260/A280 ratios between 1.8 and 2.1 were used for subsequent reverse transcription.

Complementary DNA (cDNA) was synthesized from 1–2 µg of total RNA using the ExonScript RT SuperMix with dsDNase kit (Exongen, A502). The reaction conditions were 25 °C for 10 min, 55 °C for 15 min, and 85 °C for 5 min. The resulting cDNA was stored at −20 °C until use.

qPCR was performed using SYBR Green qPCR Mix (ABI-Invitrogen, 4472920) on a Longgene Q2000B real-time PCR system. Each 20 µL reaction contained 1 µL cDNA, 10 µL 2× qPCR mix, 0.4 µL of each primer (forward and reverse), and nuclease-free water. The thermal cycling conditions were as follows: 95 °C for 5 min, followed by 40 cycles of 95 °C for 10 s, 58 °C for 20 s, and 72 °C for 20 s, followed by a melt-curve analysis to verify amplification specificity. Relative mRNA expression levels were calculated using the 2^−ΔΔCt method, with ACTIN as the internal control. The primer sequences used for qPCR were as follows:

| Gene | Direction | Sequence (5′→3′) |
| --- | --- | --- |
| Actin | F | TCCTCCTGAGCGCAAGTACTCC |
|  | R | CATACTCCTGCTTGCTGATCCAC |
| DARS2 | F | CCCAAGAGGAAGATGTGGTCC |
|  | R | AGAACAGAGTGGGGTCACG |
| PSPH | F | GACAGCACGGTCATCAGAGAAG |
|  | R | CGCTCTGTGAGAGCAGCTTTGA |
| SLC6A17 | F | TGTCGTTAAGGGCATCCAGT |
|  | R | GATGCCATCAACTGCCCCT |

1. Protein Extraction and Western Blotting (WB)

Total protein was extracted from cultured cells using RIPA lysis buffer (Beyotime, P0013C) supplemented with protease inhibitor cocktail (Beyotime, P1005). For adherent cells, cultures were washed twice with PBS, lysed on ice for 30 min, and disrupted by ultrasonication (XC-CD, Xianchang). Suspended cells were collected by centrifugation, washed with PBS, and lysed using the same method. Lysates were centrifuged at 12,000 × g for 10 min at 4 °C, and the supernatants were collected for protein quantification using the BCA Protein Assay Kit (MDL, MD913053).

Equal amounts of protein (20 µg per lane) were mixed with loading buffer, boiled at 100 °C for 10 min, and separated by 10–12% SDS-PAGE (MDL, MD911919). After electrophoresis, proteins were transferred onto 0.22 µm nitrocellulose membranes (GVS, ISEQ00010) using a wet transfer system (Junyidongfang, JY-ZY6). Membranes were blocked in 5% non-fat milk in TBST for 1 h at room temperature, then incubated overnight at 4 °C with the following primary antibodies: anti-β-actin (Affinity, AF7018, 1:3000), anti-PSPH (Proteintech, 14513-1-AP, 1:1000), anti-DARS2 (Proteintech, 13807-1-AP, 1:1000), and anti-SLC6A17 (Affinity, DF9727, 1:1000). After washing, membranes were incubated with HRP-conjugated secondary antibodies (goat anti-rabbit IgG, C030212, 1:5000) for 1 h at room temperature.

Protein bands were visualized using enhanced chemiluminescence (ECL) reagents (Clinx, ChemiScope 6100) and quantified by densitometry. β-Actin served as a loading control.

1. Construction and Validation of PSPH Knockdown and Overexpression Systems

Three siRNA sequences specifically targeting human PSPH were synthesized by Haixing Bio (China) as follows: si-1203 (5'-GGCAACAAGUCAAGGAUAA-3'), si-670 (5'-GGUCAUCAGAGAAGAAGGA-3'), and si-936 (5'-GGAGUAUUGUAGAGCAUGU-3').

A non-targeting siRNA sequence was used as a negative control. All oligonucleotides were supplied as lyophilized powders and dissolved in RNase-free water to a final concentration of 100 μM.

For overexpression, the full-length PSPH coding sequence (696 bp, NM_004577.4) was cloned into the pcDNA3.1(+) vector (KpnI–EcoRI sites) by Saierfei Biotechnology (Order No. G76052-1). The construct sequence and orientation were verified by restriction enzyme digestion and Sanger sequencing.

Transient transfection was then performed using Lipofectamine 3000 (Thermo Fisher Scientific) according to the manufacturer’s instructions. Specifically, for siRNA transfection, T24 cells were seeded to 40–50% confluence and transfected with 50 nM siRNA per well (6-well plate). For plasmid transfection, 2 μg of PSPH-pcDNA3.1(+) or empty vector DNA was used per well.

After 48 h, cells were collected for quantitative PCR and Western blot analysis to evaluate PSPH expression. The results demonstrated effective PSPH overexpression and identified si-670 as the most efficient siRNA for gene silencing.

Subsequent functional assays were conducted in both T24 and 5637 cells, with four experimental groups established as follows: untreated (UT), negative control (NC; empty vector), si-PSPH (si-670), and oe-PSPH (PSPH-overexpression plasmid). The NC group served to control for transfection-related effects.

1. Immunofluorescence Staining

Cells cultured on glass coverslips were collected 48 h after transfection for immunofluorescence staining. Both transfected and control samples were processed in parallel.

Coverslips were gently rinsed with cold PBS and fixed with 4% paraformaldehyde (Shanghai Yungan, 30525-89-4) on ice for 15 min. Fixed cells were washed twice with PBS (5 min each) and then blocked with goat serum (Beijing Yuanheng, 080608) at 37 °C for 1 h.

Subsequently, cells were incubated overnight at 4 °C with the appropriate primary antibody (PSPH, Proteintech, 14513-1-AP, 1:200) diluted in antibody dilution buffer (Boster, AR1016). After five PBS washes, samples were incubated with a fluorescent secondary antibody (goat anti-rabbit IgG [H+L], MDL, MD912533, 1:500) for 1 h at 37 °C in the dark. Nuclei were counterstained with DAPI (Beyotime, C1005) for 2 min, followed by three PBS washes.

Finally, coverslips were mounted with an anti-fade mounting medium (MDL, 30093360) and immediately examined under a fluorescence microscope (Nikon ECLIPSE CI). All procedures were carried out under minimal light exposure to preserve fluorescence intensity.

1. Wound-Healing Migration Assay

Cells were seeded into 6-well plates and grown to a confluent monolayer 48 h after transfection. A sterile 200 μL pipette tip was used to create straight scratches across the cell monolayer, guided by pre-marked reference lines on the back of the plate. Detached cells were removed by washing three times with PBS, and the medium was replaced with serum-free culture medium.

Plates were then incubated at 37 °C with 5% CO₂, and images were captured at 0 h and 48 h using an inverted microscope. The migration rate was calculated using the following formula:

$$\text{Migration rate (\%)}=\frac{W_{0}-W_{48}}{W_{0}}\times100\%$$

where $W_{0}$ and $W_{48}$ represent the wound widths at 0 h and 48 h, respectively.

1. Cell Proliferation Assay (CCK-8)

Cells were seeded into 96-well plates at a density of 1×105 cells per 100 μL per well and cultured until adherence. The four groups—UT, NC, si-PSPH, and oe-PSPH—were established, and transfection was performed accordingly. Cells were maintained at 37 °C with 5% CO₂.

At 24, 48, and 72 h after transfection, 10 μL of CCK-8 reagent (Fluorescence, DCM7122) was added to each well and incubated for 1 h. The absorbance at 450 nm was measured using a microplate reader (BIO-RAD, Model 550).

The optical density (OD) value of each group was corrected by subtracting the blank control. The relative proliferation activity was calculated using the following formula, with the UT group serving as the reference:

$$\text{Proliferation activity (\%)}=\frac{OD_{\text{group}}-OD_{\text{blank}}}{OD_{\text{UT}}-OD_{\text{blank}}}\times100\%$$

where $OD_{\text{group}}$, $OD_{\text{UT}}$, $OD_{\text{blank}}$ and represent the absorbance of the experimental groups (NC, si-PSPH, and oe-PSPH), the UT, and the blank wells, respectively.

1. Transwell Invasion Assay

Cell invasion was assessed using Matrigel-coated Transwell chambers (COStar, 3422) with 8-μm pore membranes. Matrigel was thawed at 4 °C overnight and diluted with pre-cooled serum-free medium to a final concentration of 1 mg/mL. A total of 100 μL diluted Matrigel was added to the upper chamber and incubated at 37 °C for 4–5 h to allow gel solidification.

Cells were collected 48 h after transfection, digested with trypsin (Gibco, 25200-072), washed with PBS (MDL, MD911702) and serum-free medium, and resuspended in serum-free medium at 2 × 10⁵ cells/mL. Then, 100 μL of the cell suspension was added to the upper chamber, while 600 μL of complete medium containing 10% FBS was placed in the lower chamber as a chemoattractant.

After incubation for 48 h at 37 °C with 5% CO₂, non-invaded cells on the upper surface of the membrane were gently removed with a cotton swab. The invaded cells on the lower surface were fixed with methanol (Xilong Scientific, 1710091) for 30 min and stained with crystal violet (MDL, MD911626) for 20 min at room temperature.

Membranes were rinsed with water, air-dried, and mounted on glass slides with neutral resin. The invaded cells were imaged and counted in at least three randomly selected fields under a light microscope (NOVEL XS-2100).

1. Cell Cycle and Apoptosis Analysis by Flow Cytometry

Cells from the four experimental groups—untreated control (UT), negative control (NC), si-PSPH, and oe-PSPH—were collected 48 h after transfection for cell cycle and apoptosis analyses using a flow cytometer (BENM DIAG, BeamCyte-1026). All procedures were performed according to the manufacturer’s protocols.

1. Cell Cycle Analysis

Cell cycle distribution was determined using the Cell Cycle Detection Kit (Sizhengbo Bio, FXP021). Briefly, cells were trypsinized, collected by centrifugation at 1000 rpm for 5 min, washed twice with cold PBS, and resuspended in 1 mL of PBS. Cells were then fixed in 70% pre-cooled ethanol at 4 °C for at least 2 h. After fixation, cells were washed twice with cold PBS and resuspended in propidium iodide (PI) staining solution containing RNase A, freshly prepared according to the kit instructions. Samples were incubated at 37 °C for 30 min in the dark, followed by flow cytometric analysis.

DNA content histograms were analyzed to determine the percentages of cells in the G0/G1, S, and G2/M phases using standard gating strategies based on PI fluorescence intensity.

1. Apoptosis Analysis

Cell apoptosis was evaluated using the Annexin V-FITC/PI Apoptosis Detection Kit (Sizhengbo Bio, FXP018). Cells were digested with trypsin (without EDTA), washed twice with cold PBS, and resuspended in 1× binding buffer at a final concentration of 1–5 × 10⁶ cells/mL. Then, 100 μL of the cell suspension was incubated with 5 μL Annexin V-FITC for 5 min in the dark, followed by the addition of 10 μL PI (20 μg/mL) and 400 μL PBS. Samples were analyzed immediately by flow cytometry.

The proportion of cells in each quadrant was quantified as follows: Annexin V⁻/PI⁻, viable cells; Annexin V⁺/PI⁻, early apoptotic cells; Annexin V⁺/PI⁺, late apoptotic or necrotic cells; and Annexin V⁻/PI⁺, mechanically damaged cells.

Data were processed using the instrument’s analysis software, and gating was performed to exclude debris and doublets before quantification.

1. Statistical Analysis

Statistical analyses for the cell experiments were performed using GraphPad Prism 10.6 (GraphPad Software, San Diego, CA, USA). Data are expressed as mean ± standard deviation (SD) from at least three independent experiments. One-way or two-way analysis of variance (ANOVA) was used to compare differences among groups, followed by Tukey’s multiple comparison test. Statistical significance was determined by comparing the si-PSPH and oe-PSPH groups with both the untreated control (UT) and negative control (NC) groups. The results are presented as follows: p > 0.05 indicates no significant difference (ns); p < 0.05, * or # represents significant differences compared with the UT and NC groups, respectively; p < 0.01, ** or ##; p < 0.001, *** or ###; p < 0.0001, **** or ####.

# Supplementary Figures and Tables

## Supplementary Figures

**
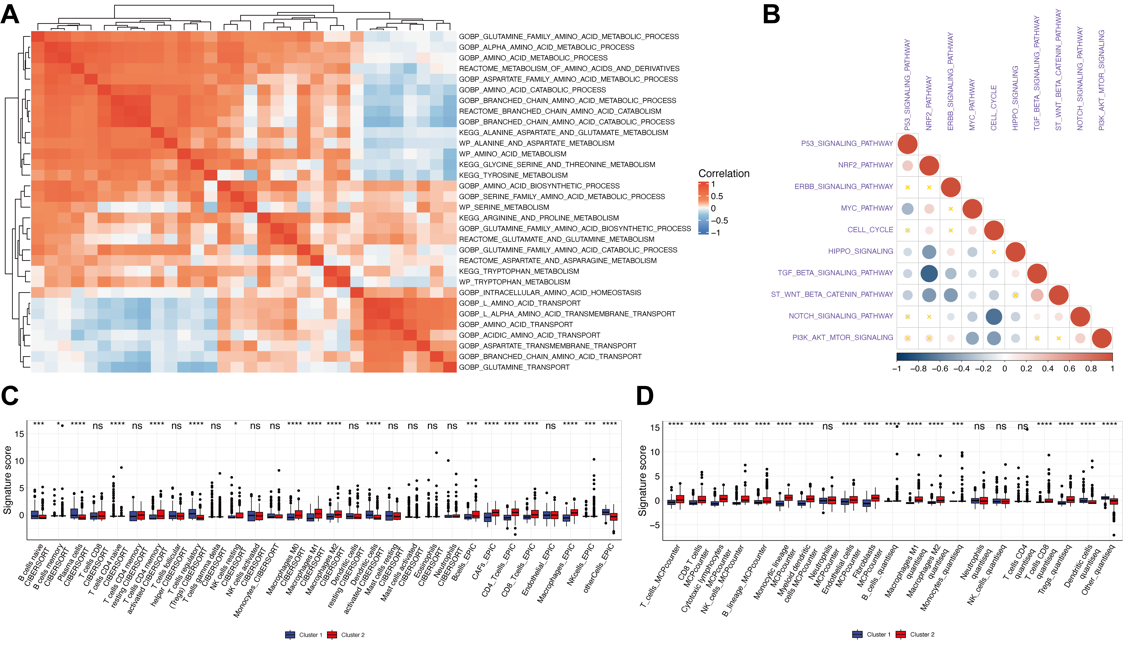
**

**Supplementary Figure1.** Pathway Correlation Analysis and Molecular Clustering of Immune Infiltration Differences. (A) Amino acid metabolism pathway correlation analysis results. (B) Correlation analysis of ten cancer-related pathways. (C-D) Immune cell level differences between molecular subtypes, as determined by CIBERSORT, EPIC, MCPcounter, and quantiseq algorithms.

**
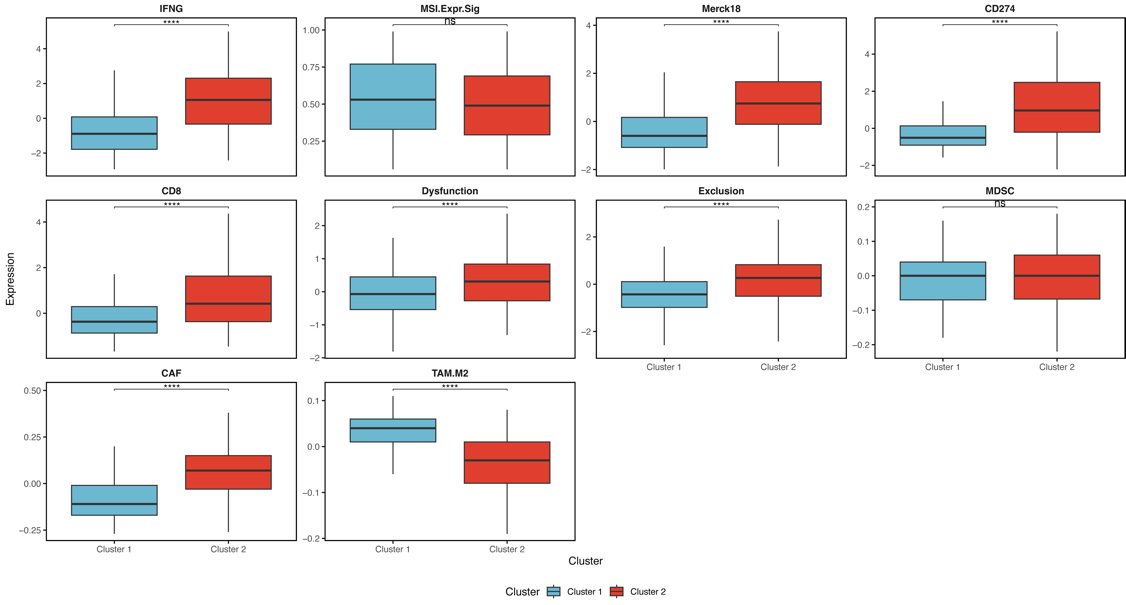
**

**Supplementary Figure2.** Differential levels of immune escape and immune exclusion between the two Clusters based on TIDE analysis.

**
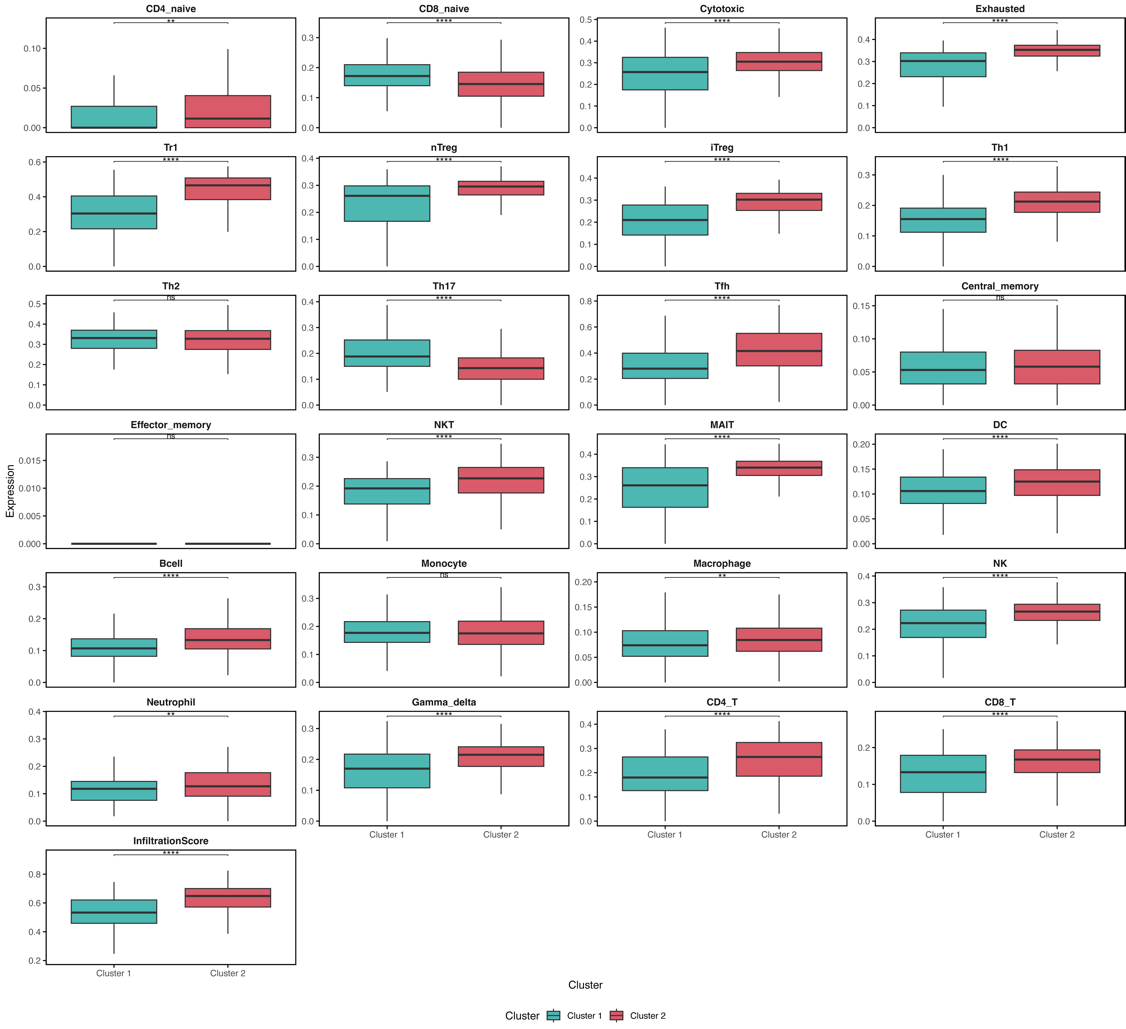
**

**Supplementary Figure3.** Immune cell level differences across Clusters based on ImmuneAI algorithm.

**
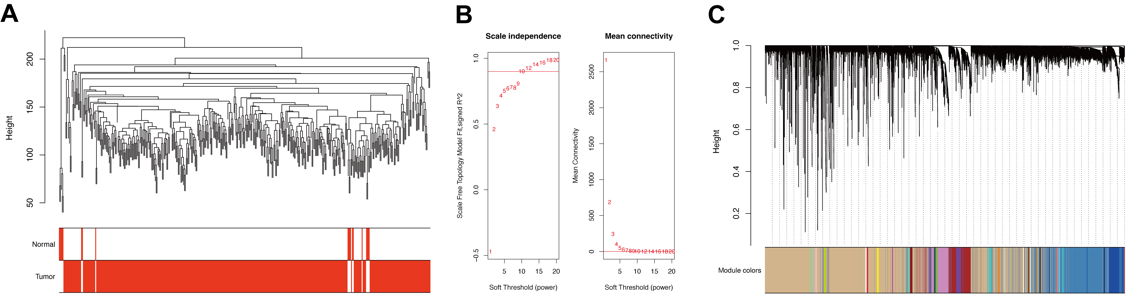
**

**Supplementary Figure4.** WGCNA analysis of gene co-expression networks: clustering (A), threshold selection (B), and module identification (C).

**
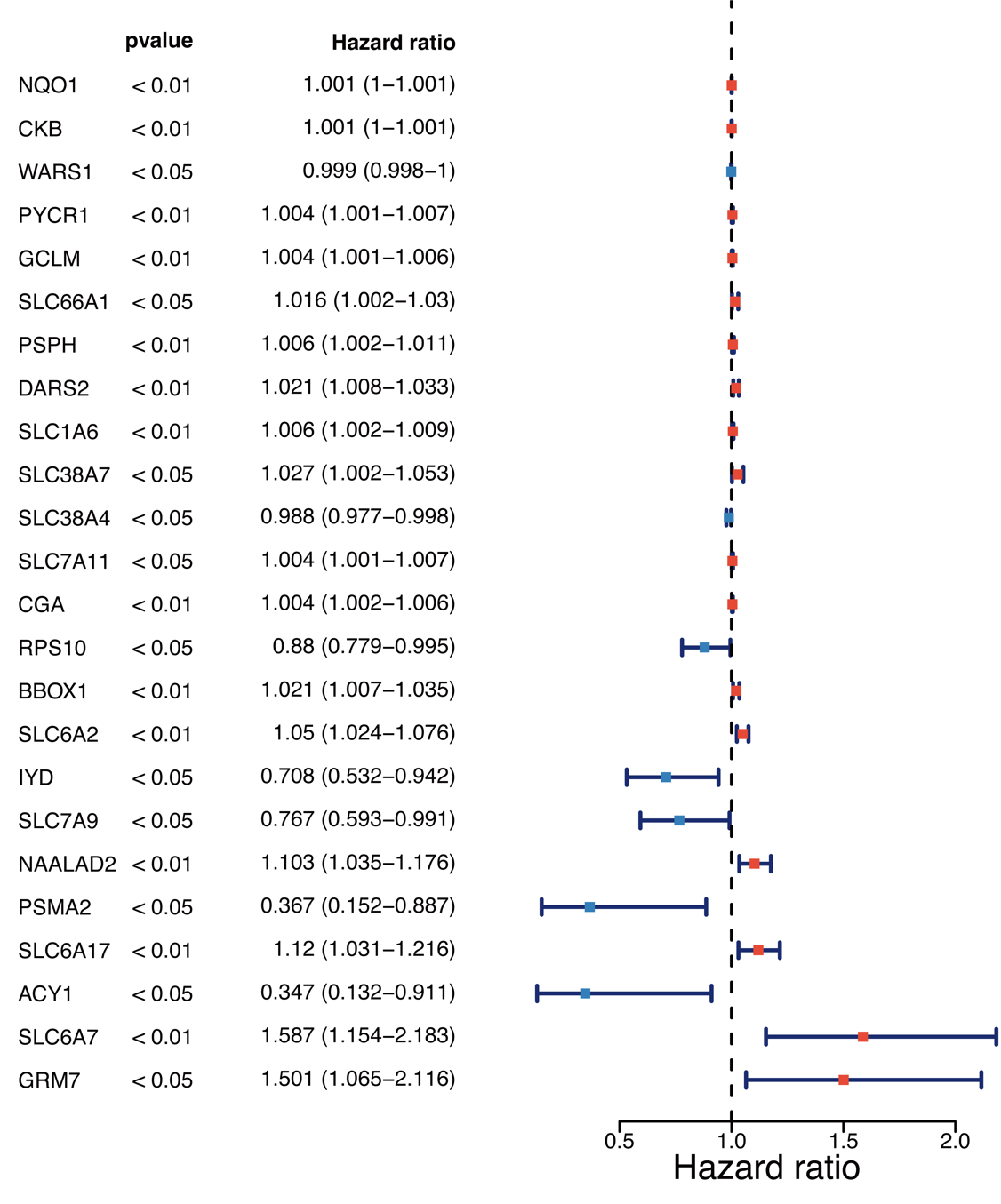
**

**Supplementary Figure5.** Results of univariate regression analysis of candidate genes.

**
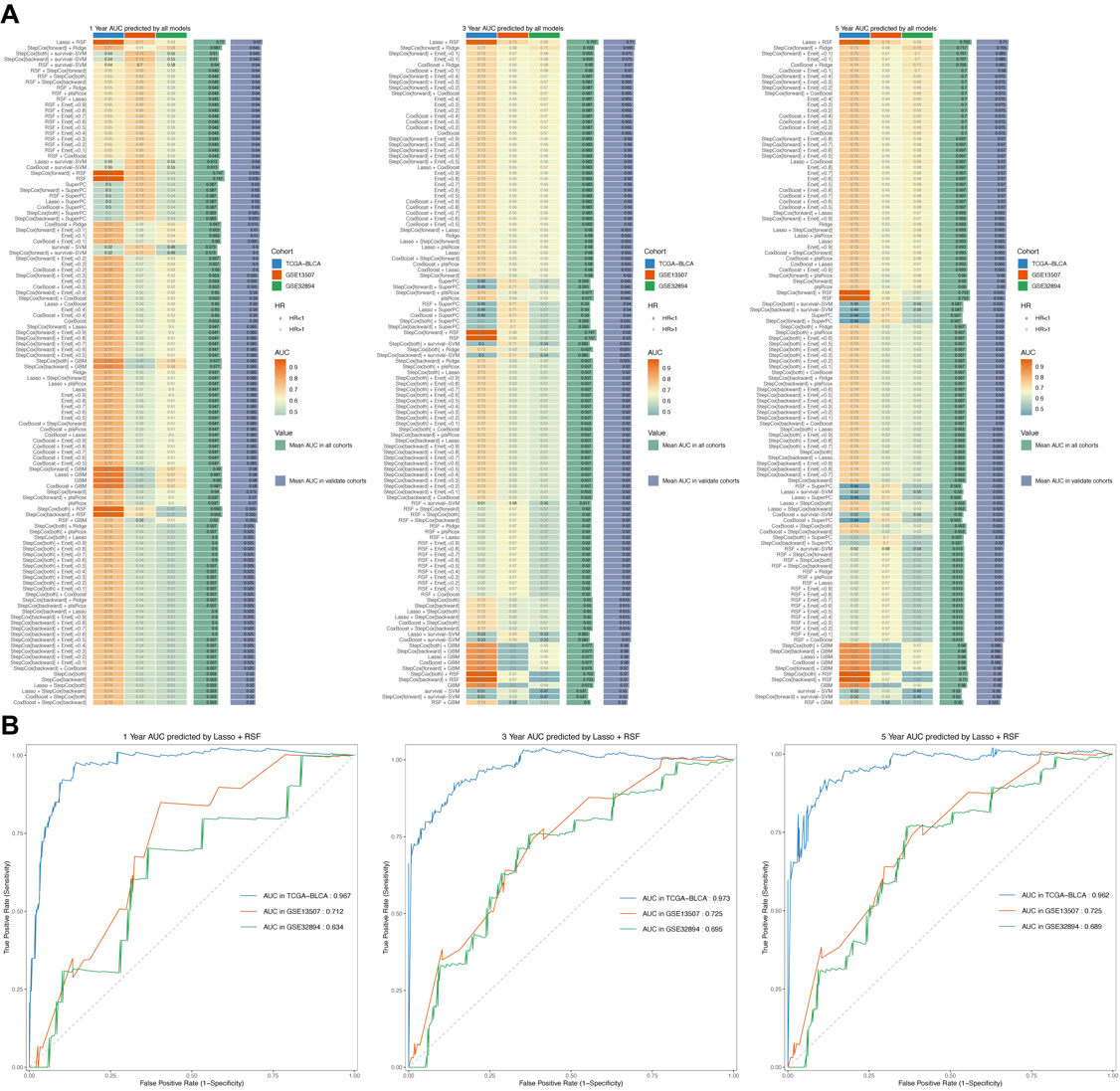
**

**Supplementary Figure6.** Prognostic model AUC results at 1, 3, and 5 years in the training cohort (TCGA-BLCA) and validation cohorts (GSE13507 and GSE32894). (A) Differences in the 117 algorithm combinations. (B) AUC results from LASSO + RSF.

**
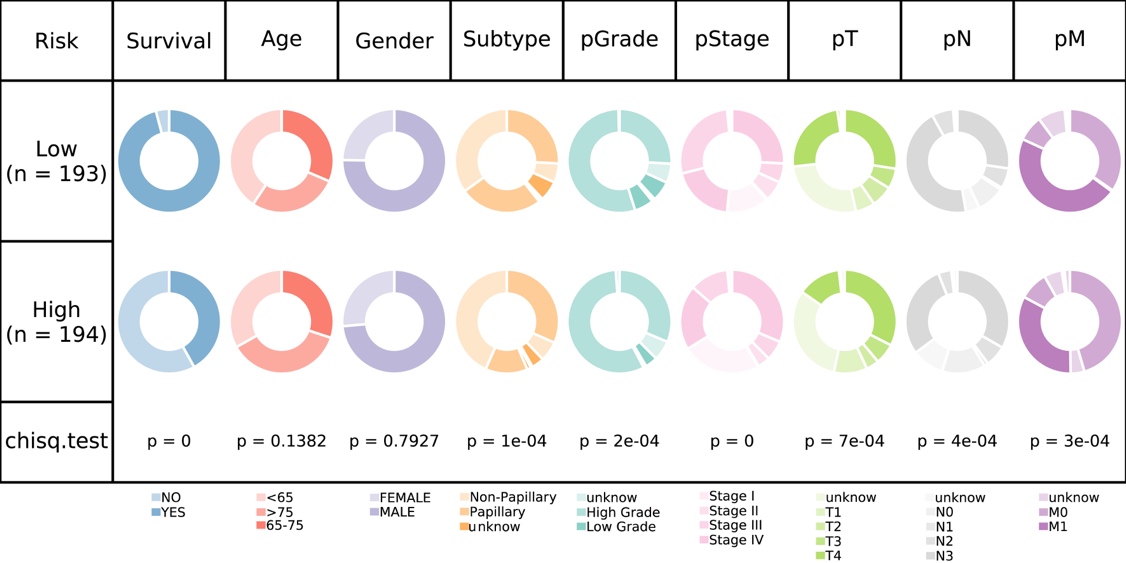
**

**Supplementary Figure7.** Comparison of Clinical Characteristics Across Different Risk Groups.

**
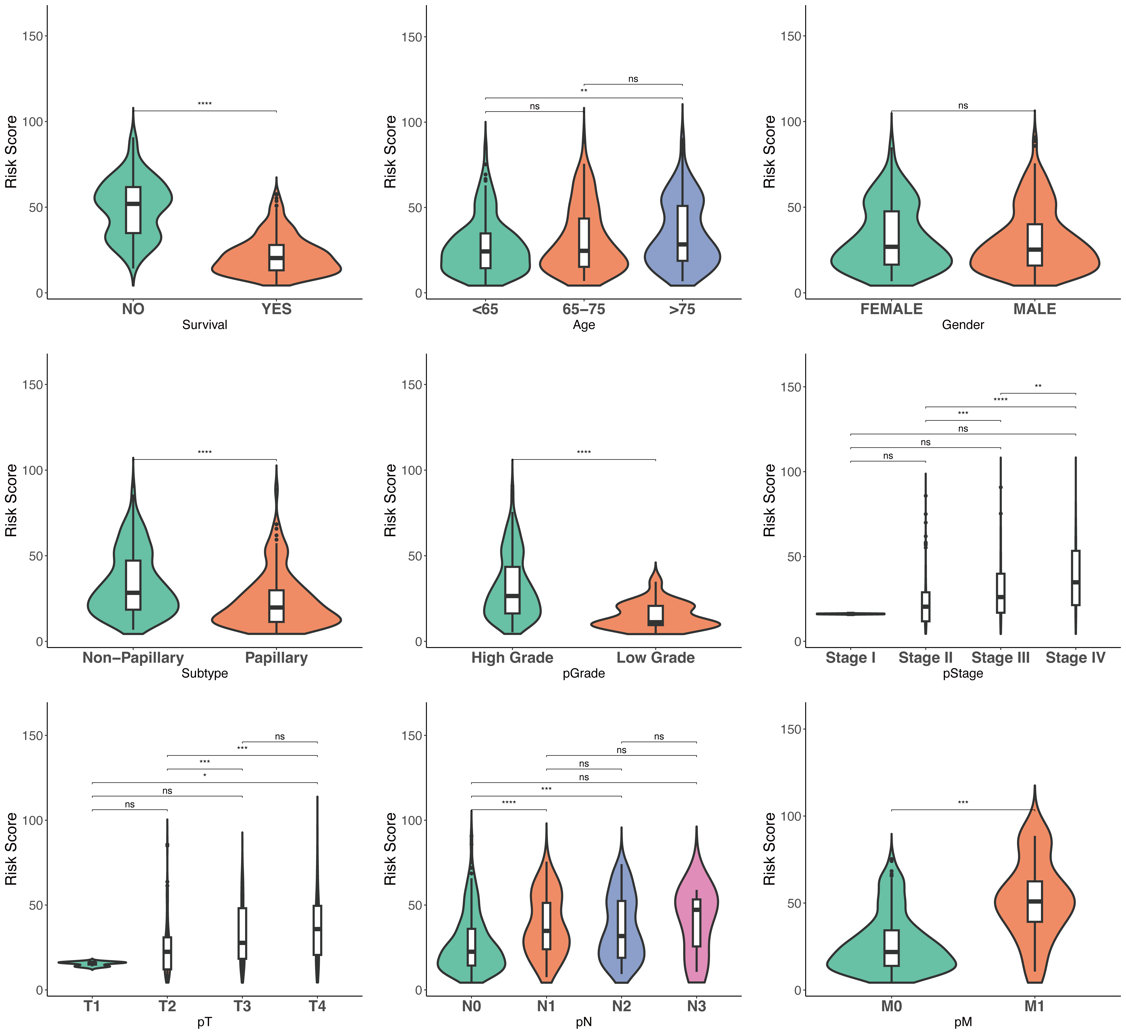
**

**Supplementary Figure8.** Differences in Risk Scores Across Groups with Different Clinical Characteristics.

**
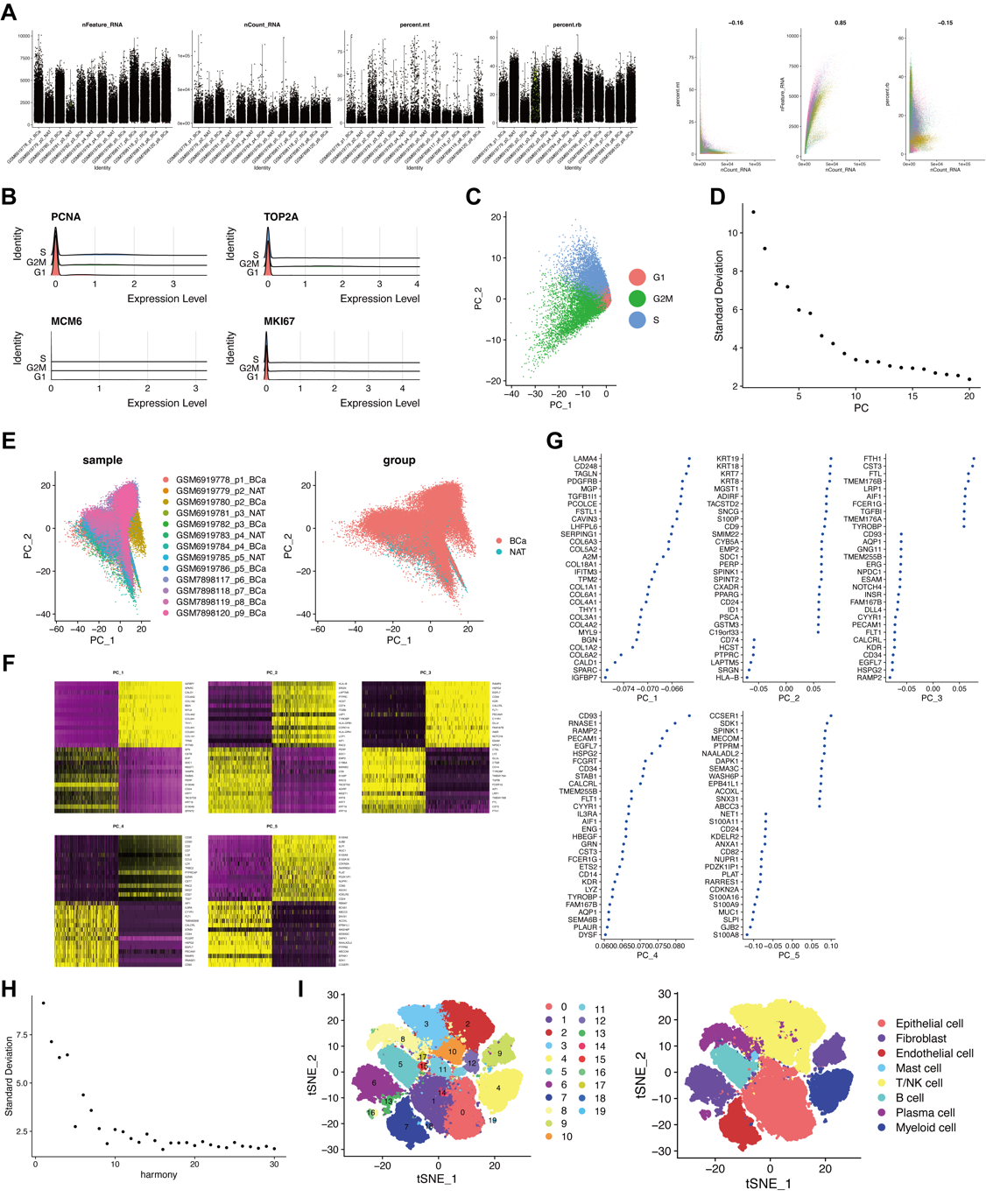
**

**Supplementary Figure9.** Preprocessing of Single-Cell RNA Sequencing Data (GSE222315). (A) Quality control filtering based on feature count, total transcript count, mitochondrial gene expression, and ribosomal gene expression. (B) Expression distribution of cell cycle markers (e.g., PCNA, TOP2A) across different cell cycle phases (G1, S, G2M). (C) Principal component analysis (PCA) based on cell cycle marker expression, showing the distribution of cell cycle phases (G1, S, G2M) in the PC1 and PC2 dimensions. (D) Variance proportion analysis of principal components to determine the components retained for subsequent analysis. (E) Distribution of samples and groups in principal component space to observe inter-sample variability. (F) Gene expression patterns for the top five principal components (PC1–PC5). (G) Variance proportion changes after Harmony integration of principal components. (H) Distribution of 19 cell clusters in t-SNE space based on Seurat clustering (resolution 0.5). (I) Annotation of cell clusters to specific cell types using known marker genes, and visualization of cell type distribution in t-SNE space.

**
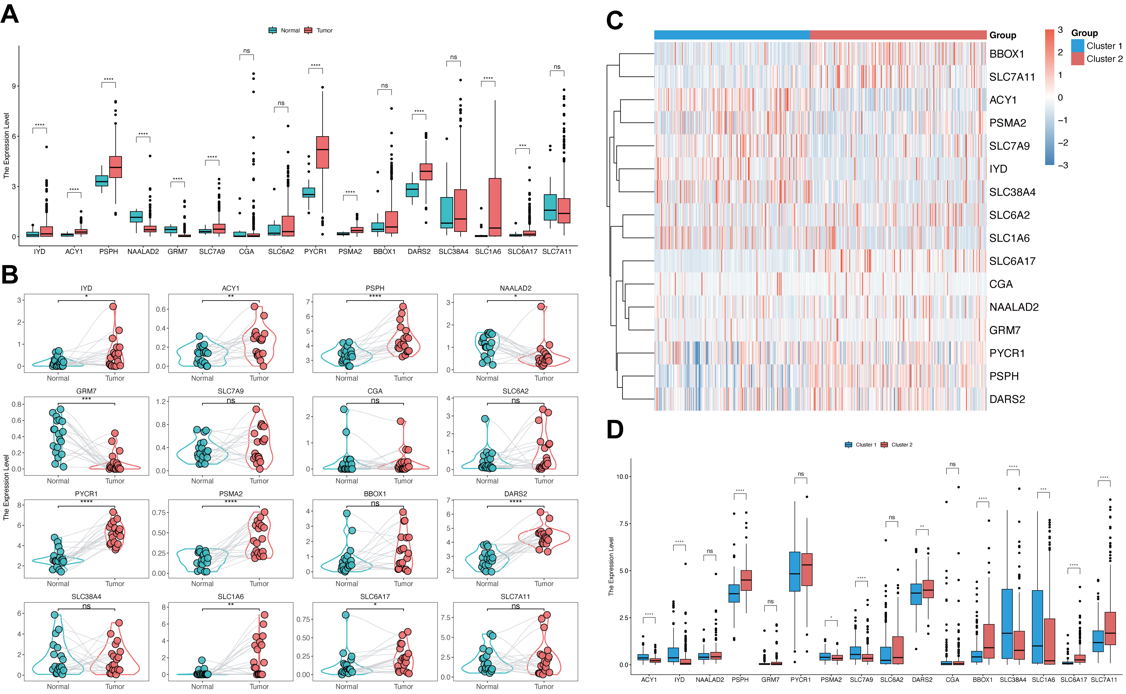
**

**Supplementary Figure10.** Differential Analysis of Prognostic Model Genes Between Tumor and Adjacent Normal Tissues, and Across Different Molecular Subtypes. (A) Unpaired differential analysis between tumor and adjacent normal samples. (B) Paired differential analysis between tumor and adjacent normal samples. (C-D) Expression differences of model genes across different subtypes.

**
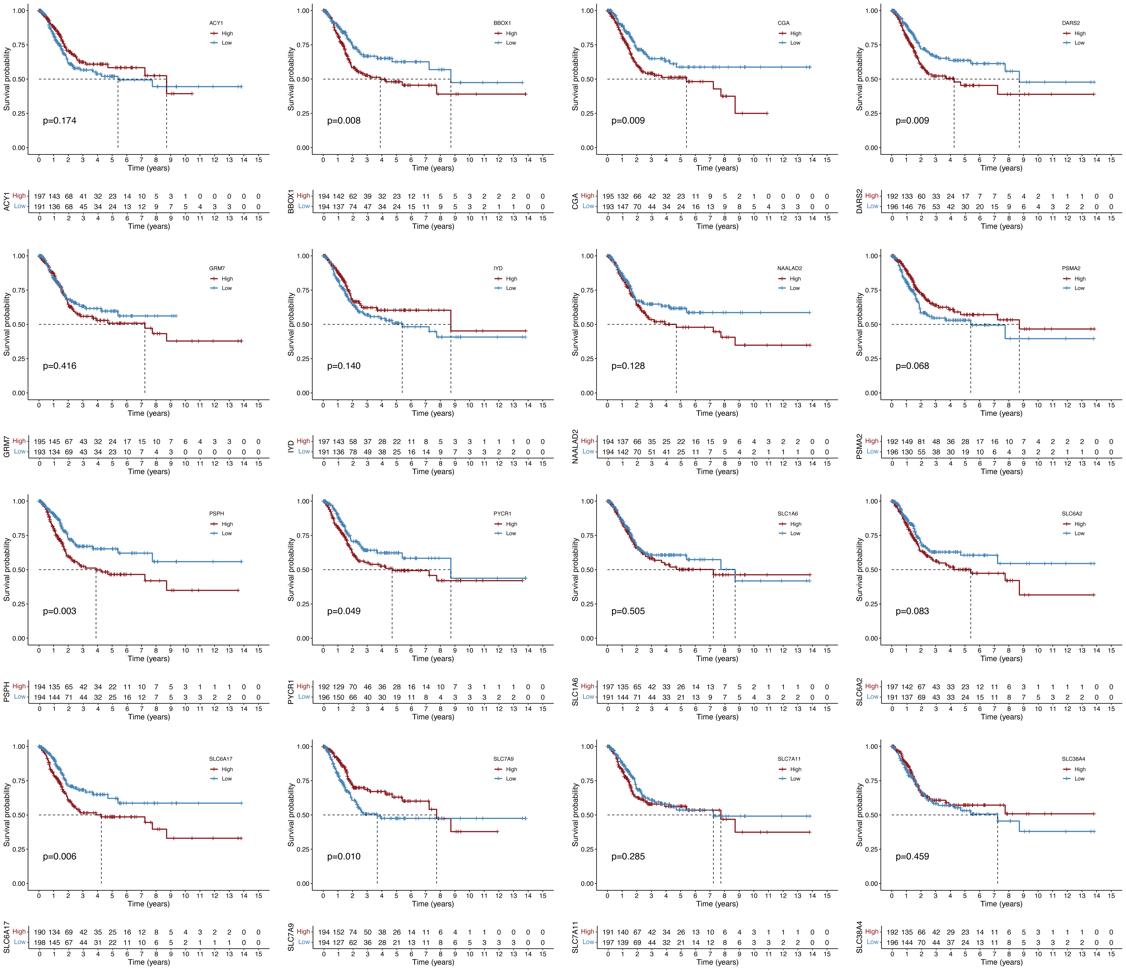
**

**Supplementary Figure11.** Survival difference analysis of model genes, stratified by high and low expression based on the gene expression median.

**
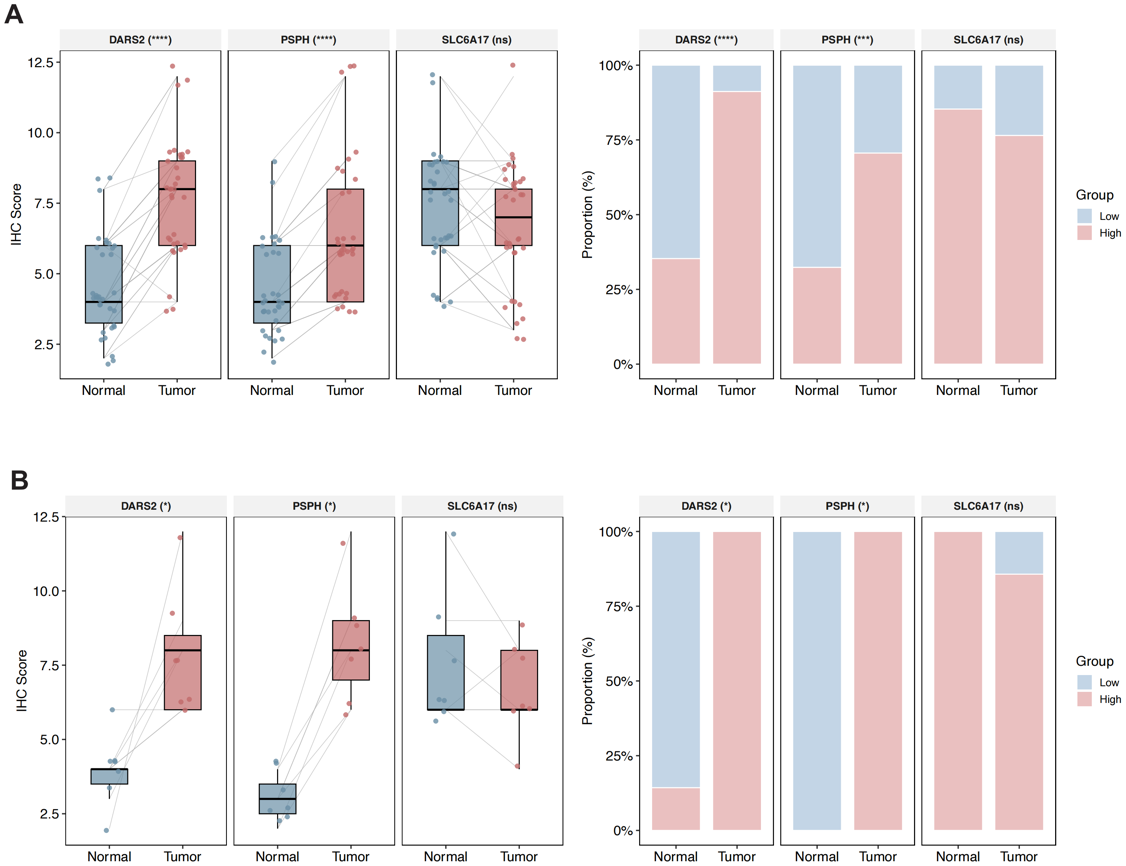
**

**Supplementary Figure12.** Statistical analysis of PSPH immunohistochemical staining in high-grade and low-grade urothelial carcinoma of the bladder. (A) Paired statistical evaluation of PSPH immunohistochemistry in high-grade urothelial carcinoma, including continuous scoring data (left) and categorical immunoreactivity analysis (right). (B) Paired statistical evaluation of PSPH immunohistochemistry in low-grade urothelial carcinoma based on continuous staining scores (left) and corresponding categorical data (right).

**
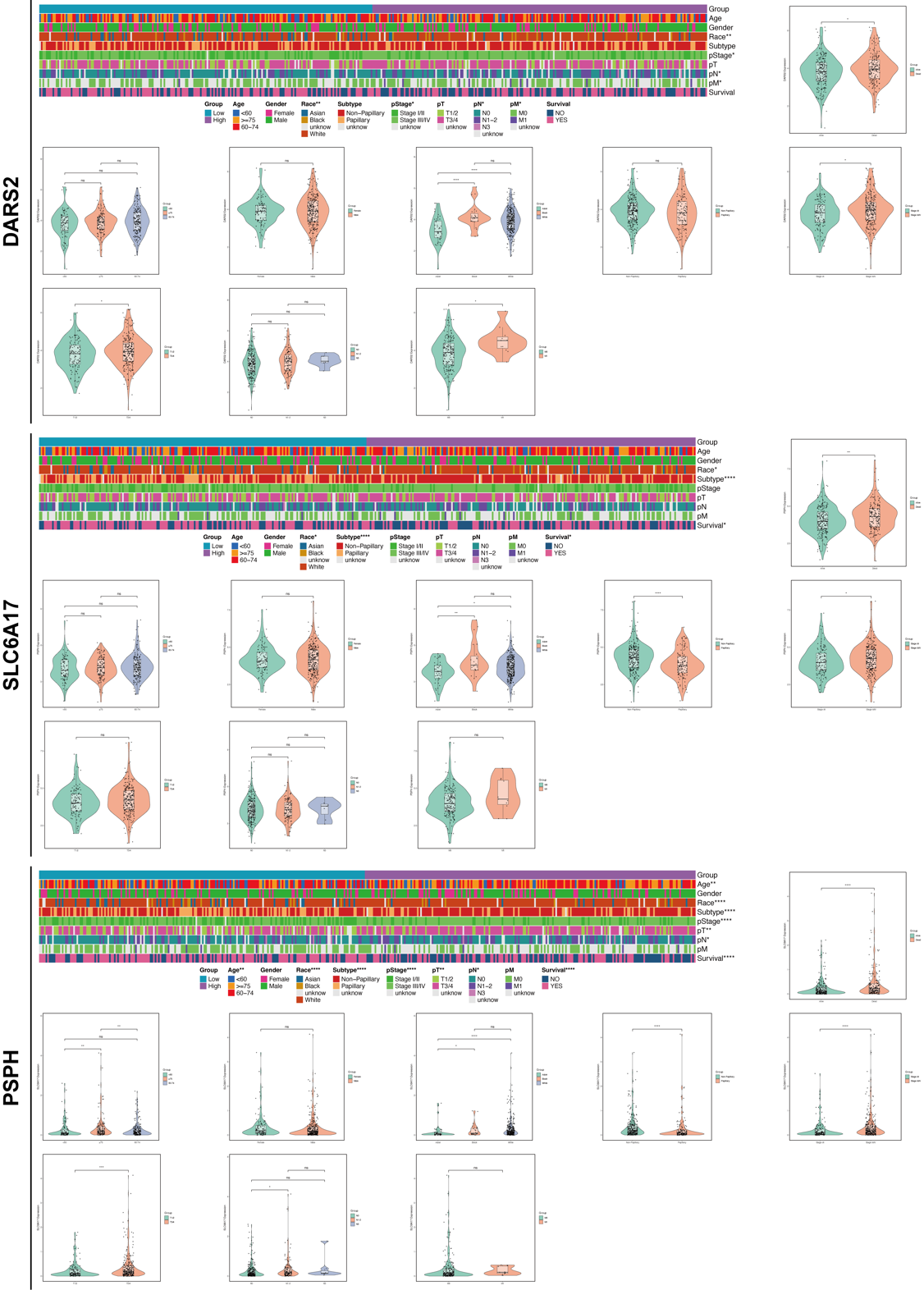
**

**Supplementary Figure13.** Association of DARS2, PSPH, and SLC6A17 expression with clinicopathological features in TCGA-BLCA cohort. (A) Clinical correlation analysis of DARS2 expression in 404 TCGA-BLCA patients. Gene expression was dichotomized by median value for group comparisons, and continuous-variable analyses were performed to assess associations with clinicopathological characteristics. (B) Clinical correlation analysis of PSPH expression using the same cohort and analytical approach, including median-based grouping and continuous-scale statistical testing. (C) Clinical correlation analysis of SLC6A17 expression, showing its relationship with major clinicopathological parameters based on both grouped comparisons and continuous-data evaluation.


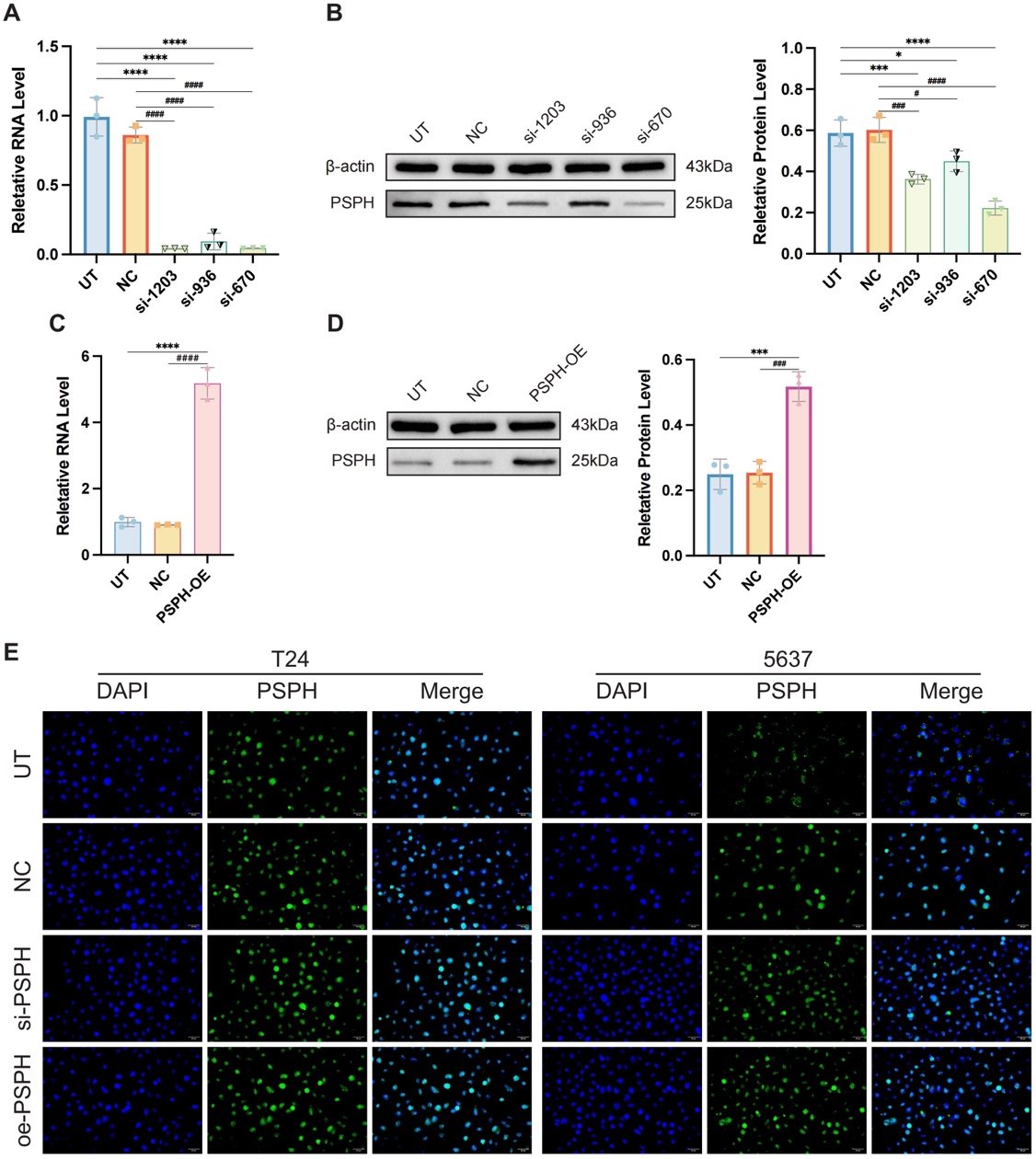


**Supplementary Figure14.** Validation of PSPH knockdown and overexpression in T24 cells and immunofluorescence staining in bladder cancer cell lines. (A–B) qPCR and Western blot analyses showing the knockdown efficiency of three siRNAs targeting PSPH (si-1203, si-936, si-670) in T24 cells relative to untreated (UT) and negative control (NC) groups. Western blot panels include representative bands and densitometric quantification. si-670 was used in subsequent experiments. (C–D) qPCR and Western blot confirmation of PSPH overexpression in T24 cells transfected with the PSPH-OE plasmid, with UT and NC serving as controls. Western blot panels show representative bands and corresponding quantification. (E) Immunofluorescence staining (100×) of PSPH in T24 and 5637 cells across UT, NC, si-PSPH (si-670), and oe-PSPH groups, illustrating expression changes after knockdown or overexpression.

## Supplementary Tables

The Supplementary Tables associated with this study are provided as Supplementary Tables 1–3. Each supplementary file comprises several tables; readers are advised to refer to the individual files for detailed information.
